# MiR393 Regulation of Auxin Signaling and Redox-Related Components during Acclimation to Salinity in Arabidopsis

**DOI:** 10.1371/journal.pone.0107678

**Published:** 2014-09-15

**Authors:** María José Iglesias, María Cecilia Terrile, David Windels, María Cristina Lombardo, Carlos Guillermo Bartoli, Franck Vazquez, Mark Estelle, Claudia Anahí Casalongué

**Affiliations:** 1 Instituto de Investigaciones Biológicas, UE-CONICET-UNMDP, Facultad de Ciencias Exactas y Naturales, Universidad Nacional de Mar del Plata, Mar del Plata, Argentina; 2 Departamento de Biología e Instituto de Investigaciones Biológicas, UE-CONICET-UNMDP, Facultad de Ciencias Exactas y Naturales, Universidad Nacional de Mar del Plata, Mar del Plata, Argentina; 3 Botanical Institute of the University of Basel, Zürich-Basel Plant Science Center, Part of the Swiss Plant Science Web, Department of Environmental Sciences, Basel, Switzerland; 4 Instituto de Fisiología Vegetal, Facultad de Ciencias Naturales, Universidad Nacional de La Plata-CCT La Plata CONICET, La Plata, Argentina; 5 Section of Cell and Developmental Biology, University of California San Diego, San Diego, California, United States of America; 6 Howard Hughes Medical Institute, University of California San Diego, San Diego, California, United States of America; Iwate University, Japan

## Abstract

One of the most striking aspects of plant plasticity is the modulation of development in response to environmental changes. Plant growth and development largely depend on the phytohormone auxin that exerts its function through a partially redundant family of F-box receptors, the TIR1-AFBs. We have previously reported that the Arabidopsis double mutant *tir1 afb2* is more tolerant to salt stress than wild-type plants and we hypothesized that down-regulation of auxin signaling might be part of Arabidopsis acclimation to salinity. In this work, we show that NaCl-mediated salt stress induces miR393 expression by enhancing the transcription of *AtMIR393A* and leads to a concomitant reduction in the levels of the TIR1 and AFB2 receptors. Consequently, NaCl triggers stabilization of Aux/IAA repressors leading to down-regulation of auxin signaling. Further, we report that miR393 is likely involved in repression of lateral root (LR) initiation, emergence and elongation during salinity, since the *mir393ab* mutant shows reduced inhibition of emergent and mature LR number and length upon NaCl-treatment. Additionally, *mir393ab* mutant plants have increased levels of reactive oxygen species (ROS) in LRs, and reduced ascorbate peroxidase (APX) enzymatic activity compared with wild-type plants during salinity. Thus, miR393 regulation of the TIR1 and AFB2 receptors could be a critical checkpoint between auxin signaling and specfic redox-associated components in order to coordinate tissue and time-specific growth responses and tolerance during acclimation to salinity in Arabidopsis.

## Introduction

Soil salinity is one of the most significant abiotic stresses to crop productivity worldwide [1]. Therefore, elucidation of plant acclimation to salinity has become a main issue in plant physiology. The rapid perception of salinity and accurate relay of environmental signals to switch on adaptive developmental responses are essential for plant survival under conditions of high salinity. In this context, auxin, which is an established growth regulator, is emerging as a new player in plant responses to biotic and abiotic stresses [2], [3].

The natural auxin, indole-3-acetic acid (IAA) modulates gene expression through direct physical interaction with the TIR1/AFBs auxin receptor (TAAR) proteins resulting in the targeted degradation of Aux/IAA transcriptional repressor proteins via the SCF E3-ubiquitin ligase proteasome pathway [4], [5], [6], [7], [8]. Recent findings demonstrated that TAARs have distinct biochemical and biological functions and exhibit a complex post-transcriptional regulation [9], [10], [11]. *MiR393* and the secondary siRNAs have been implicated in down-regulation of TAARs expression in Arabidopsis [11], [12], [13], [14], [15].

Reactive oxygen species (ROS) and hormones are key elements in intricate switches used by plants to trigger highly dynamic responses to changing environment. Although ROS may have deleterious effects in the cells, they also act as signal transduction molecules involved in mediating responses to environmental stresses [16]. Plant plasticity in response to the environment is linked to a complex signaling module in which ROS and antioxidants operate together with hormones, including auxin [17]. We previously reported the involvement of TAARs in the plant adaptive response to oxidative and salinity stresses. The auxin resistant double mutant *tir1 afb2* showed increased tolerance to salinity measured by chlorophyll content, germination rate and root elongation. In addition, mutant plants displayed reduced hydrogen peroxide (H_2_O_2_) and superoxide anion (O_2_^−.^) levels, as well as enhanced antioxidant metabolism [18]. Microarray analyses indicated that auxin responsive genes are repressed by different stresses such as, wounding, oxidative, selenium, and salt treatments in *Arabidopsis* and rice [19], [20], [21]. More recently, the transcriptomic data of Blomster et al. [22] showed that various aspects of auxin homeostasis and signaling are modified by apoplastic ROS. Together, these findings suggest that the suppression of auxin signaling might be a strategy that plants use to enhance their tolerance to abiotic stress including salinity. However, whether auxin signaling is repressed as a result of salt stress and how stress-related signals and plant development are integrated by a ROS-auxin crosstalk is still in its beginning.

Here, we show that salinity triggers miR393 expression which leads to a repression of TIR1 and AFB2 receptors. Furthermore, down-regulation of auxin signaling by miR393 was demonstrated to mediate the repression of LR initiation, emergence and elongation during salinity. Additionally, the *mir393ab* mutant showed increased levels of reactive oxygen species (ROS) due to reduced ascorbate peroxidase (APX) enzymatic activity. Altogether these experiments lead us to propose a hypothetical model to explain how salt stress might suppress TIR1/AFB2-mediated auxin signaling thus integrating stress signals, redox state and physiological growth responses during acclimation to salinity in Arabidopsis plants.

## Materials and Methods

### Plant Material and Growth Conditions

*Arabidopsis thaliana* wild-type (WT) and mutants *tir1-1* [23], *tir1-1 afb2-3* [24], *ago1-27* [25] and *mir393ab* [26], are in the Columbia (Col-0) ecotype. Arabidopsis transgenic lines *BA3pro:GUS*, *DR5pro:GUS*, *HSpro:AXR3NT-GUS*, *TIR1pro:TIR1-GUS*, *TIR1pro:mTIR1-GUS*, *AtMIR393Apro:GUS*, *AtMIR393Bpro:GUS*, *35Spro:TIR1-Myc, mir393ab HSpro:AXR3NT-GUS and mir393ab DR5pro:GUS* were previously described [4], [9], [26], [27], [28], [29].

Seeds were surface-sterilized and stratified at 4°C in the dark for 2 d. Then, seeds were plated on *Arabidopsis thaliana* Salt (ATS) medium [30] plus 1% (w/v) sucrose and 0.8% (w/v) agar and grown vertically at 23°C under 120 µmol photons m^−2^ s^−1^ with 16∶ 8 h light: dark cycles.

For lateral roots (LRs) assays, 4 days post-germination (dpg) seedlings growing on ATS agar medium on vertical plates were transferred onto fresh media containing NaCl for additional 5–7 d, after which the number of emergent and mature LR was measured according to Malamy and Benfey [31]. Alternatively, 4 dpg seedlings were transferred from auxin-free medium onto 85 nM indole acetic acid (IAA) or 85 nM 2,4-D and LR were counted after 3 d as previously described by Dharmasiri et al. [6]. Since IAA, is susceptible to photolysis under blue and ultraviolet light, for IAA-treatment plants were grown under yellow light as described by Rahman et al. [32].

For rosette diameter measurements, plants were grown in ATS medium supplemented with 75 mm NaCl in horizontal position for 12 d. Rosette area was determined by finding the minimal area that contained all leaves using ImageJ as image-analysis software (http://rsbweb.nih.gov/ij/).

Unless stated otherwise, seedlings were grown on ATS medium in vertical position and then transferred to liquid ATS medium supplemented with NaCl for designated times.

### GUS Staining

Transgenic lines were transferred into liquid ATS medium containing NaCl or IAA and then incubated with mild shaking at 23°C for 2–4 h. After treatment, seedlings were fixed in 90% (v/v) acetone at 20°C for 1 h, washed twice in 50 mM sodium phosphate buffer pH 7.0 and incubated in staining buffer [50 mM Na phosphate (pH 7.0), 5 mM EDTA, 0.1% (v/v) Triton X-100, 5 mM K_4_Fe(CN)_6_, 0.5 mM K_3_Fe (CN)_6_ and 1 mg/ml X-Gluc (GBT)] at 37°C from 2 h to overnight. Bright-field images were taken using a Nikon SMZ800 magnifier.

Specifically, *HSpro:AXR3NT-GUS* seedlings were induced in liquid ATS medium at 37°C for 2 h and then treated with NaCl at 23°C.

For the analysis of GUS expression in cross sections of primary roots, seedlings were included in a paraffin matrix (Paraplast) at 60°C after GUS staining. Roots were cut into 5 µm sections using a Minot type rotary microtome Zeiss HYRAX M 15. Section were deparaffined with xylene, mounted with Entellan (Merk) and observed by bright field microscopy in an Olympus CX21 microscope. Images were captured using a digital camera attached to the microscope. The arrangement of cells in the cross section of primary roots was evaluated according to Malamy and Benfey [31]. Densitometric analysis of GUS expression was conducted by scanning blue vs total pixels of the different tissues using Matrox Inspector 2.2 software (Matrox Electronics Systems, Ltd). The control value was arbitrarily set to 1 in each case.

### Protein Gel Blot Analysis

Seven dpg *tir1-1 35S:TIR1-Myc* plants were transferred to liquid ATS medium supplemented with 200 mM NaCl for different times. After treatments, plants were homogenized in ice-cold buffer [50 mM 2-amino-2-(hydroxymethyl)-1,3-propanediol (TRIS) pH 7.5, 200 mM NaCl, 10% (v/v) glycerol, 0.1% (v/v) Tween-20], containing 1 mM phenylmethylsulfonyl fluoride (PMSF) and complete protease inhibitor cocktail (Roche) and centrifuged twice at 10,000 g at 4°C for 15 min. Equal amounts of protein were loaded onto SDS-PAGE and blotted onto the nitrocellulose membrane. Membranes were incubated with anti-c Myc antibody (Sigma) and goat anti-mouse alkaline phosphatase conjugate (Sigma) was used as secondary antibody. Then, Myc detection was visualized with NBT and BCIP (Promega). Densitometry analysis of immunoblots was performed using Matrox Inspector 2.2 software (Matrox Electronics Systems, Ltd).

### RNA Preparation and RNA-analysis

Total RNA was isolated using TRIzol reagent (Invitrogen). RNA samples were assessed for purity via their *A*_260_/*A*_280_ ratios and integrity by resolving 1 µg of total RNA on a 1.2% (w/v) denaturing agarose gel. For each sample, a normalization of RNA for RT was performed by density measurement of each 28S ribosomal RNA band. Total RNA (1 µg) was reverse transcribed with ImProm-II Reverse Transcription System (Promega) following the manufacturer's protocol. The level of transcript was determined by RT-PCR using the following primers: *TIR1* (TIR1-F 5′-GATGGTCTCGCTGCTATCG-3′ and TIR1-R 5′-GGTTGAAGCAAGCACCTCA-3′), *AFB2* (AFB2-F 5′-CTGCCAACAAATGACAAACG-3′ and AFB2-R 5′-ATCCATTCTTGTCCCAACCA-3′), *GUS* (GUS-F 5′-GGCACAGCACATCAAAGAGA-3′ and GUS-R 5′-CTGATAGCGCGTGACAAAAA-3′), *ACT2* (ACT-F 5′-AAACCCTCGTAGATTGGCACA-3′ and ACT2-R 5′-AAACCCTCGTAGATTGGCACA-3′). Conditions were optimized for all semi-quantitative RT-PCR reactions to ensure linearity of response for comparison between samples.

RNA-blots were done according to Si-Ammour et al. [33].

### Chlorophyll Content

Seven dpg seedlings were transferred into liquid ATS medium supplemented with 100 mM NaCl during 3 d and chlorophyll was extracted as described in detail previously [18]. The chlorophyll content was measured spectrophotometrically at 652 nm (UltrospecTM 1100) [34].

### In situ ROS detection

Seedlings were incubated with 10 µM of the cell permeable fluorescent probe 2′,7′-dicloro-dihydro fluorescein (H2DCF DA, Sigma-Aldrich) in 5 mM MES Buffer pH 5.7, 250 µM ClK and 1 mM Cl_2_Ca during 30 min in darkness. After three washes, seedlings were examined by epi-fluorescence in an Eclipse E200 microscope (Nikon, http://www.nikon.com/) connected with a high-resolution digital camera (Nikon). Fluorescence intensity in LRs was quantified using ImageJ as image-analysis software (http://rsbweb.nih.gov/ij/).

H2DCF DA is de-esterified intracellularly and turns to highly fluorescent 2′,7′-dichlorofluorescein upon oxidation.

### Superoxide Detection

To assay superoxide anion (O_2_^.−^) leaves from 14 dpg WT and *miR393ab* seedlings were stained with 0.2% (w/v) NBT in 10 mM potassium phosphate buffer pH 7.5 for 30 min as described by Jabs et al. [35]. Leaves were bleached in 96% (v/v) ethanol overnight.

### ROS Measurements

Seven dpg seedlings were transferred into liquid ATS medium supplemented with 100 mM NaCl for 12 h and H_2_O_2_ was extracted as described in detail previously [18]. Endogenous H_2_O_2_ was quantified based on the reaction of xylenol orange (*o*-cresolsulfonephthalein 3′,3″-bis (methylimino) diacetic acid sodium salt with the peroxide-mediated oxidation of Fe^2+^ to Fe^3+^ [36].

### APX and CAT Activity

Seven dpg seedlings were transferred to liquid ATS medium supplemented with 100 mM NaCl for 12 h. Catalase (CAT) and ascorbate peroxidase (APX) activities were measured as described in detail previously [18]. Total proteins were measured according to Bradford [37] by using bovine serum albumin as standard.

### Ascorbate and GSH measurements

Seedlings (0.5–1 g) were ground in liquid N_2_ and the powder was extracted in 6% trifluoracetic acid (TFA) followed by centrifugation at 13,000 g for 5 min. AA level was measured by high performance liquid chromatography (HPLC) as described in detail previously [38].

GSH was measured in the same homogenates used for AA determinations. Total thiols were assayed spectrophotometrically in a reaction mixture containing 100 mM K_2_HPO_4_ buffer pH 7.5, 5 mM EDTA, 0.5 U mL^−1^ glutathione reductase, 0.5 mM 5,5′-dithiobis-(2-nitrobenzoic acid), 0.1 mM NADPH and different sample volumes [39]. GSSG was determined after treating samples with 2-vinylpiridine.

### Statistical Analysis

The values shown in figures are mean values ± SE of at least 3 experiments. Approximately, 50 seedlings were processed per line in each experiment. The data were subjected to analysis of *t*-test or variance (one and two- ways ANOVA) and post hoc comparisons were performed with Tukey's multiple range test at *P*<0.05 level. SigmaStat 3.1(SPSS) was used as statistical software program.

## Results

### Auxin-dependent Physiological Responses in Whole Seedlings are Affected by Salinity

**Figure 1:**
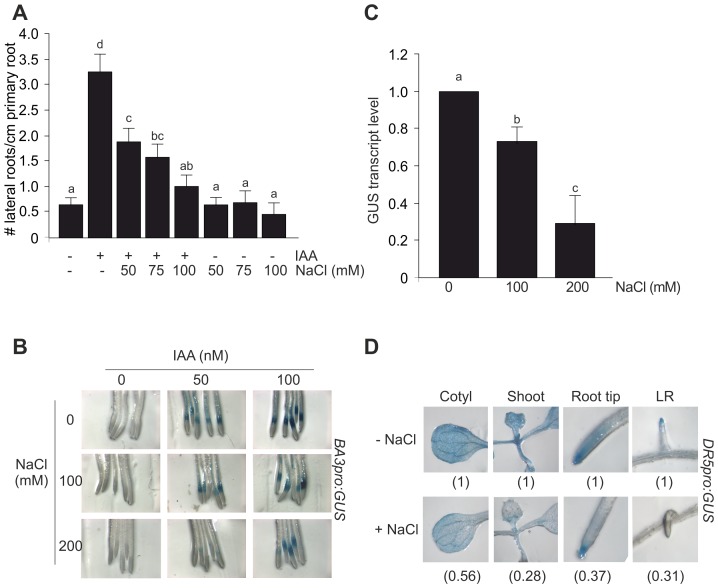
Auxin responses are affected by salinity in Arabidopsis seedlings. (**A**) Four dpg WT seedlings were transferred from auxin-free medium onto ATS medium containing no auxin or 85 nM IAA in combination with increasing concentrations of NaCl. The total number of emerged lateral roots was counted 4 d after the transfer to new media. Data are mean values (±SE) of three independent experiments. Different letters indicate a significant difference at P≤0.05 (Tukey test). (**B**) Seven dpg *BA3pro:GUS* seedlings were incubated on 50 nM or 100 nM IAA in combination with increasing concentrations of NaCl for 4 h. GUS activity was revealed after incubation with X-Gluc at 37°C. GUS staining in representative root tip segment is shown. (**C**) Relative transcript level of GUS upon 100 nM IAA treatment in combination with NaCl as described in (B). The control value is arbitrarily set to 1 in each case. Data are mean values (±SE) of three independent experiments. Different letters indicate a significant difference at P≤0.05 (Tukey test). (**D**) Seven dpg *DR5pro:GUS* seedlings were incubated with 200 mM NaCl for 4 h and subjected to GUS staining. Images show different parts of seedlings: cotyledons (Cotyl), shoots, root tip and LR. The GUS signal detected in NaCl treatment relative to control is shown. The control value is arbitrarily set to 1 in each case. Data are mean values of three independent experiments.

The induction of LRs represents a very rapid, sensitive and quantitative parameter to evaluate an auxin-mediated response [40]. To explore the regulation of auxin-dependent physiological responses by salt, four dpg seedlings were transferred from auxin-free medium onto media containing IAA or the synthetic auxin 2,4-D in combination with increasing concentrations of NaCl (50, 75 and 100 mM). After 3 d, LRs were quantified. As shown in Figure 1A and Figure S1, NaCl inhibited auxin-mediated induction of LRs in a dose-response manner suggesting that salinity treatment counteracts auxin-dependent growth responses in Arabidopsis plants. This conclusion was also confirmed by exploring the effect of salinity on the inducibility of the auxin signaling reporters *BA3pro:GUS* and *DR5pro:GUS* seedlings. To induce auxin response, 7 dpg seedlings were transferred to liquid ATS medium containing 50 nM or 100 nM IAA in combination with increasing concentrations of NaCl (100 and 200 mM) for 4 h. IAA treatments stimulated *GUS* expression in a dose-dependent manner as indicated by a bright-blue colour in root tips of *BA3pro:GUS* seedlings, NaCl had a clear inhibitory effect in auxin-mediated *GUS* expression (Figure 1B). In 100 nM IAA-treated *BA3pro:GUS* seedlings, GUS mRNA level was quantified under increasing concentrations of NaCl. Approximately, a 75% repression in auxin response was estimated through GUS mRNA level quantification in 200 mM NaCl-treated seedlings compared with control (Figure 1C). In agreement with this result, NaCl treatment blocked GUS expression in whole *DR5pro: GUS* seedlings including cotyledons, shoots, root tips, and LR (Figure 1D). These results showed a correlation between the formation of less LR (Figure 1A) and the reduction of auxin-dependent promoter inducibility in LRs by NaCl.

### Salinity Represses TIR1/AFB2-Mediated Auxin Signaling

**Figure 2:**
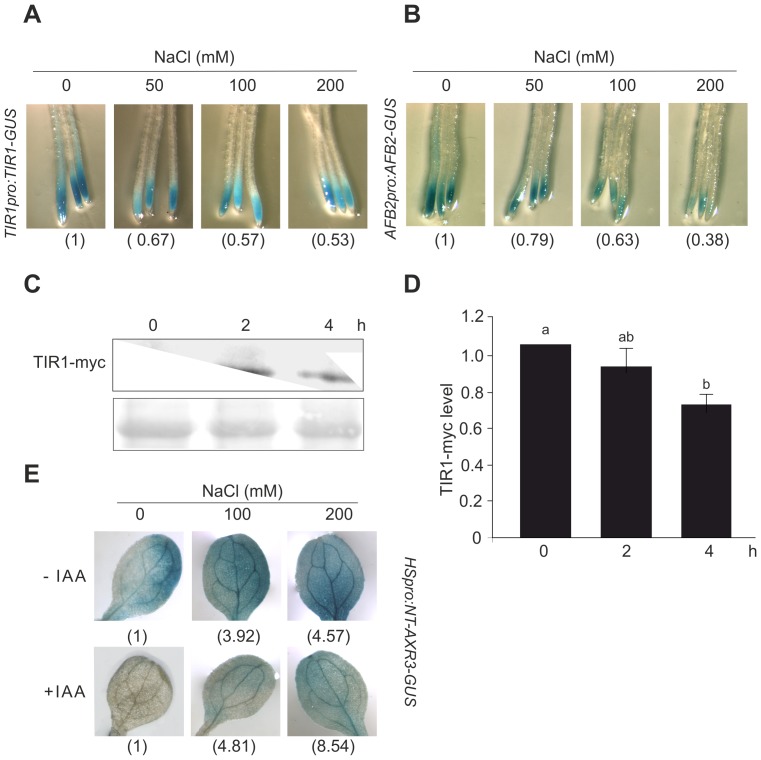
Salinity represses auxin signaling pathway. Seven dpg *TIR1pro:TIR1-GUS* (**A**) and *AFB2pro:AFB2-GUS* (**B**) seedlings were incubated in liquid ATS medium with increasing concentrations of NaCl for 4 h and then subjected to GUS staining. Representative photographs of root tips are shown. The control value is arbitrarily set to 1 in each case. Data are mean values of three independent experiments. (**C**) Seven dpg *tir1-1 35S::TIR1-Myc* plants were transferred to liquid ATS medium supplemented with 200 mM NaCl for different times. Total proteins were extracted and protein blot analysis was carried out using an anti cMyc antibody (upper panel). Ponceau staining of Rubisco is shown (bottom panel). (**D**) Densitometric analysis of three independent immunoblots as indicated in (C). Data are mean values (±SE). Different letters indicate a significant difference at P≤0.05 (Tukey test). (**E**) Seven dpg *HSpro:AXR3NT-GUS* seedlings were transferred for 2 h to 37°C and then incubated with 100 and 200 mM NaCl in the absence (upper panel) or presence (lower panel) of IAA. GUS activity was revealed after incubation with X-Gluc at 37°C. Representative photographs and the GUS signal detected in NaCl treatment relative to control is shown. The control value is arbitrarily set to 1 in each case. Data are mean values of three independent experiments.

NaCl might inhibit auxin response by modulation of auxin signaling. Auxin-mediated gene expression is regulated via TAARs. Thus, NaCl action may result in transcriptional repression of *TAAR* genes. To test this possibility, the effect of NaCl-mediated salinity was analysed in *TIR1pro:TIR1-GUS* and *AFB2pro:AFB2-GUS* seedlings. In both transgenic lines, GUS enzyme is fused to the C-terminal region of the TIR1 or AFB2 protein and expressed under the control of their respective natural promoters. Firstly, we explored TIR1 level in *TIR1pro:TIR1-GUS* seedlings after NaCl treatment in a time-course assay. Four hours of NaCl treatment was sufficient to down regulate TIR1 level (Figure S2C). Then, 7 dpg *TIR1pro:TIR1-GUS* and *AFB2pro:AFB2-GUS* seedlings were subjected to increasing concentrations of NaCl (50 mM, 100 mM and 200 mM) for 4 h. As shown in Figure 2, A and B, NaCl reduced GUS staining in both lines suggesting a reduction in TIR1 and AFB2 protein levels by salt. *AFB2pro:AFB2-GUS* line showed a dose-depend inhibition of AFB2 content while NaCl at 50 mM was sufficient to reduce and restrict GUS staining to the root tip in *TIR1pro:TIR1-GUS* line. In agreement, Arabidopsis plants expressing a cMyc epitope-tagged TIR1 under the *CaMV 35S* promoter showed a reduction of approximately 30% of TIR1 protein level in whole seedling after 4 h of 200 mM NaCl treatment (Figure 2, C and D).

In the presence of auxin, TAARs interact with Aux/IAA proteins to promote their degradation. Thus, a reduction in TIR1 and AFB2 levels should lead to less Aux/IAAs degradation. To test whether salt stress leads to stabilization of Aux/IAA proteins, we analyzed the expression of the reporter protein AXR3NT-GUS under salt treatment. The *HSpro:AXR3NT-GUS* reporter encodes a fusion between the amino terminus of the Aux/IAA repressor AXR3/IAA17 and GUS driven by a heat-shock (HS)-inducible promoter [4]. After heat shock treatment, 7 dpg seedlings were treated with IAA in combination with NaCl for 4 h and then assessed for *GUS* expression. As expected, IAA treatments caused a decrease in AXR3NT-GUS stability but this was substantially counteracted by 100 and 200 mM NaCl since seedlings exposed to salinity exhibited stronger GUS staining (Figure 2E, lower panel). The increased AXR3NT-GUS stability was also detected in NaCl-treated seedlings in the absence of IAA (Figure 2E, upper panel) suggesting that down-regulation of TIR1 and AFB2 by salinity is concomitant with the stabilization of Aux/IAA repressors in Arabidopsis leaves.

### Down-regulation of the TIR1 and AFB2 Receptors by miR393

**Figure 3:**
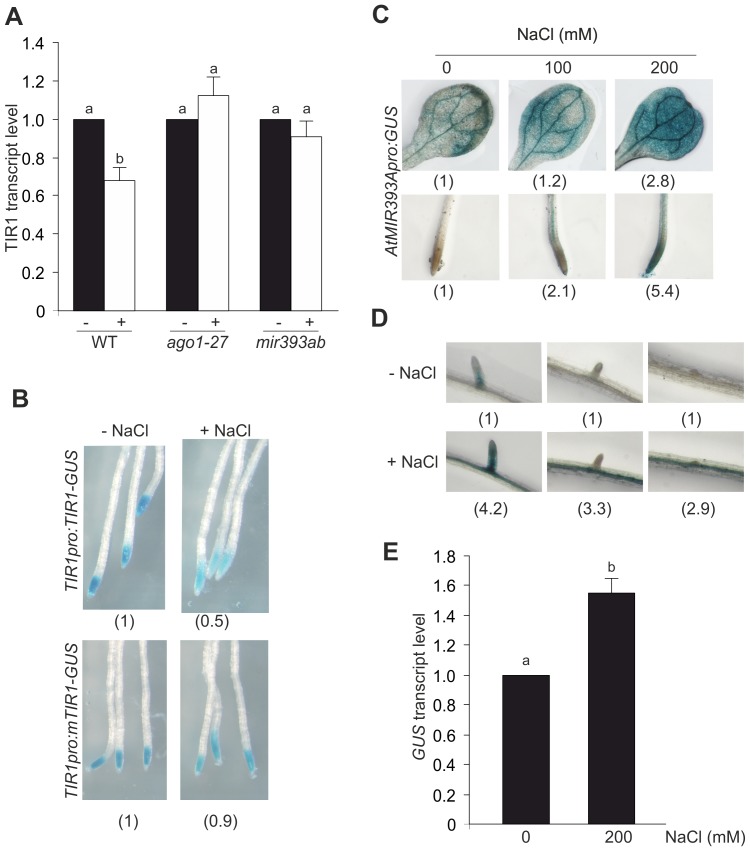
Salt-mediated down-regulation of TIR1 by miRNAs. (**A**) Seven dpg WT, *ago1-27* and *mir393ab* seedlings were subjected to 200 mM NaCl treatment for 4 h. Relative transcript level of *TIR1* upon treatment was measured by RT-PCR. The control value is arbitrarily set to 1 in each case. Data are mean values (±SE) of three independent experiments. Different letters indicate a significant difference at P≤0.05 (Tukey test). (**B**) Seven dpg *TIR1pro:TIR1-GUS* and *TIR1pro:mTIR1-GUS* seedlings were incubated in liquid ATS medium with NaCl for 4 h and then subjected to GUS staining. Representative photographs of root tips are shown. The control value is arbitrarily set to 1 in each case. Data are mean values of three independent experiments. (**C,D**) Seven dpg *AtMIR393Apro:GUS* seedlings were transferred to liquid ATS medium supplemented with increasing concentrations of NaCl for 2 h. GUS activity was revealed after incubation with X-Gluc at 37°C. GUS staining in representative leaves and root segments are shown. The control value is arbitrarily set to 1 in each case. Data are mean values of three independent experiments. (**E**) Relative transcript level of GUS in NaCl- treated *AtMIR393Apro:GUS* seedlings was quantified upon treatment. The control value is arbitrarily set to 1 in each case. Data are mean values (±SE) of three independent experiments. Different letters indicate a significant difference at P≤0.05 (Tukey test).

TIR1-mediated auxin signaling is post-transcriptionally regulated by miR393 and *siTAARs* in Arabidopsis seedlings grown in standard conditions [9], [11], [15]. Additionally, miR393 also plays important roles during responses to various stress conditions [12], [13], [14], [41]. We hypothesized that miRNA-mediated regulation of TAARs could take part during Arabidopsis adaptive response to salinity. ARGONAUTE (AGO) proteins are essential components in the RNA silencing pathways that mediate mRNA degradation or translation inhibition through the binding of small RNAs at their target sites [42]. We analysed the expression of *TIR1* in 7 dpg WT and *ago1-27* seedlings subjected to 200 mM NaCl treatment for 4 h. Whereas NaCl-mediated salinity triggered a 25% reduction of TIR1 transcript level in WT plants, *ago1-27* did not show changes after 4 h of initial treatment suggesting miRNA-mediated regulation of *TIR1* (Figure 3A). Similarly, *AFB2* also showed down-regulation by salt (Figure S3). To determine whether miR393 plays a role in TAAR regulation during salinity, TIR1 level during salinity was evaluated in *TIR1pro:mTIR1-GUS* seedlings. This line includes four silent nucleotide changes within the miR393 recognition site predicted to make the transgene resistant to miR393-directed regulation. Coherently with miR393 regulation, mTIR1 did not show changes after salt treatment in this transgenic seedling (Figure 3B). In particular, the Arabidopsis genome contains two miR393 precursors on chromosomes 2 (*AtMIR393A*) and 3 (*AtMIR393B*), both producing identical mature miR393 [43], [44]. In *MIR393pro:GUS* fusion lines, 2.5 kb upstream of each gene (*AtMIR393A* and *AtMIR393B*) is used to drive expression of the *GUS* reporter. Thus, these lines were used to visualize miR393 expression in specific tissues and determine the potential contribution of each miR393 precursor under salinity. Seven dpg *MIR393A::GUS* and *MIR393B::GUS* seedlings were treated with 200 mM NaCl for 0, 2, 4 and 6 h and then stained for GUS activity. The activation of *MIR393A* promoter was observed after 2 h of initial treatment (Figure S2A). Up-regulation of miR393 levels dependent on salt concentration was detected in shoots as well as in roots when *MIR393Apro:GUS* seedlings were subjected to increasing concentrations of NaCl for 2 h (Figure 3C). In addition, *MIR393Apro:GUS* salt-treated roots showed increase GUS intensity in the central stele of LRs including the pericycle layer (Figure 3D and Figure S4). An increase of approximately 50% of GUS mRNA level was observed in 200 mM NaCl-treated *MIR393Apro:GUS* transgenic seedlings respect to control condition (Figure 3E) which was consistent with GUS staining data (Figure 3C). However, *MIR393B* promoter was just slightly activated and GUS mRNA level was unaltered in 200 mM NaCl-treated *MIR393Bpro:GUS* seedlings suggesting that *MIR393A* is the promoter mainly induced during salinity (Figure S2B; Figures S5, A and B). The inability of *mir393ab* mutants to reduce TIR1 transcript level and to stabilize Aux/IAA repressors in NaCl-treated seedlings reinforced the role of miR393 as a regulator of TAAR during salinity (Figure 3A, Figure S6). Furthermore, quantification of miR393 by Northern blot assay indicated that miR393 level is induced 20% in roots of NaCl-treated seedlings after 4 h of treatment (Figure S7).

### Auxin-Dependent Morphological Responses are Affected by Salinity in *mir393* Mutants

**Figure 4:**
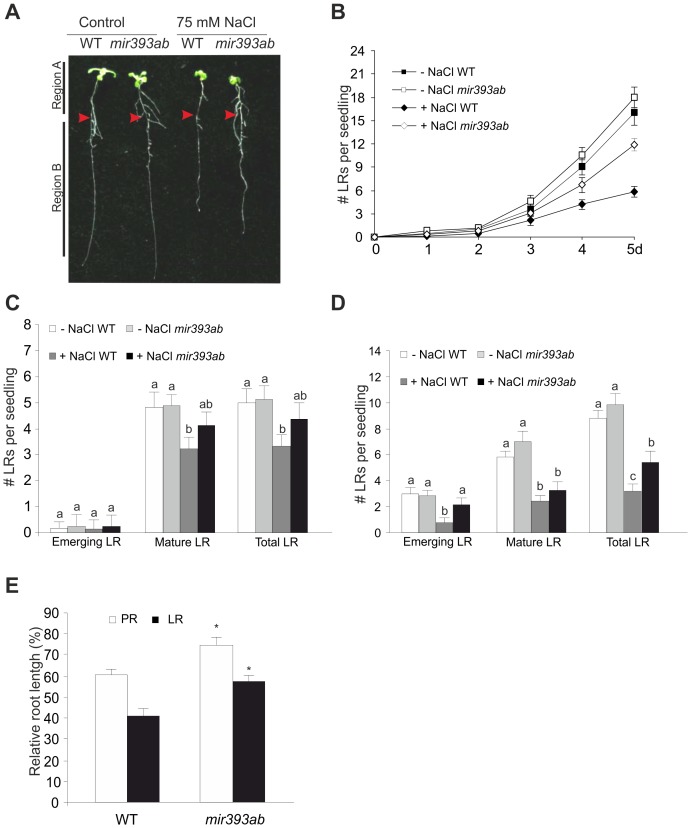
NaCl influences auxin-dependent morphological responses through miR393 regulation. Four-dpg WT and *mir393ab* seedlings were transferred onto ATS medium containing 75 mM NaCl. Arrowheads mark the position of the root tip at the time of transfer from standard to treatment conditions. Representative photographs of seedlings after 5 d of treatment are shown in (**A**) LR were quantified at designed times (**B**) Data are mean values (±SE) of three independent experiments. (**C**) and (**D**) Quantification of emergent and mature LR in root region A and region B after transfer to standard or salt stress conditions, respectively. Data are mean values (±SE) of three independent experiments. Different letters indicate a significant difference at P≤0.05 (Tukey test). (**E**) Suppression of LR and PR growth under 75 mM NaCl conditions measured as a percentage of growth relative to standard conditions. Seedlings were transferred to salt at 4 dpg and grown for 5 d post treatment. Data are mean values (±SE) of three independent experiments. Asterisks mark significant changes between WT and *mir393ab* at P≤0.05 (*t*- test). PR: WT −NaCl 7.35 cm+/−0.55; WT +NaCl 4.34 cm+/−0.39; *mir393ab* −NaCl 7.41 cm+/−0.29; *mir393ab* +NaCl 5.5 cm+/−0.17. LR: WT −NaCl 0.95 cm+/−0.15; WT +NaCl 0.39 cm+/−0.12; *mir393ab* −NaCl 0.96 cm+/−0.14; *mir393ab* +NaCl 0.57 cm+/−0.08…

The induction of miR393 during salinity with the concomitant reduction in TIR1 and AFB2 levels prompted us to determine whether miR393-mediated down-regulation of auxin signaling is involved in morphological changes that take place in seedlings growing under salt stress. Since salt treatment over 100 mM NaCl is a very strong stress that severely affects root growth, we set up a 75 mM NaCl concentration for all physiological experiments. Four dpg seedlings were transferred to medium containing NaCl and emerged LR were counted for several days. A reduction in the number of LR was observed in NaCl-treated seedlings of *mir393ab* mutant and WT after 5 d of treatment. However, *mir393ab* seedlings exhibited a lower reduction in the number of LR (28% at 75 mM NaCl) under salinity compared to WT (60%) (Figure 4, A and B). In contrast, *tir1 afb2* showed an intrinsic reduction in the LR number that was almost not affected by salt (Figure S8A and B).

Evidence from genetic and physiological studies suggest that auxin is required at different specific developmental stages to mediate LR formation, since it drives primordium formation, LR initiation, emergence and elongation [45]. In order to understand the regulatory mechanism affected in *mir393ab* mutant, root architecture was examined in more detail according to Duan et al. [46]. When 4 dpg seedlings are transferred to 75 mM NaCl treatment for additional days, two regions are established along the PR: region A (for above transfer point), where LR patterning is expected to have occurred before treatment of roots with high salinity, and region B (for below transfer point), where LR patterning occurs under the effect of NaCl. Then, we quantified the number of emergent and mature LR in both regions under control and salinity treatment according to Malamy and Benfey [31]. *mir393ab* plants showed no differences in the number of emergent LR in region A while it presented a slight increase in the number of mature LR in NaCl treatment (Figure 4C). However the higher number of LRs previously observed in *mir393ab* mutant (Figure 4B) agrees with the 2-fold higher number of emergent LRs in region B compared with WT (Figure 4D). Further studies to determine whether miR393 is involved in the increase of branch points specified along the PR or in the enhanced initiation and emergence rate will be the subject of another study and for this reason is not discussed further here. Finally, measurement of post-emergence LR length revealed that miR393 could be also involved in repression of LR elongation during salinity since *mir393ab* mutant showed a reduced inhibition (18%) of LR length in NaCl-treated seedlings compared to WT (Figure 4E). PR inhibition during salinity was also measured and although it was of less magnitude compared with the effect previously observed in LRs, again *mir393ab* mutant showed a reduced inhibition compared to WT seedlings (15%; Figure 4E). In contrast, *tir1 afb2* showed enhanced inhibition of PR growth by salt treatment (Figure S8C).

In addition, a smaller rosette size was observed when WT seedlings grow in 75 mM NaCl- treatment. However, while WT seedlings showed a reduction of 33% in the rosette area, *mir393ab* seedlings evidenced lower inhibition (Figure S9).

### miR393 Regulation of Auxin Signaling Triggers Changes in Redox Related Components

**Figure 5:**
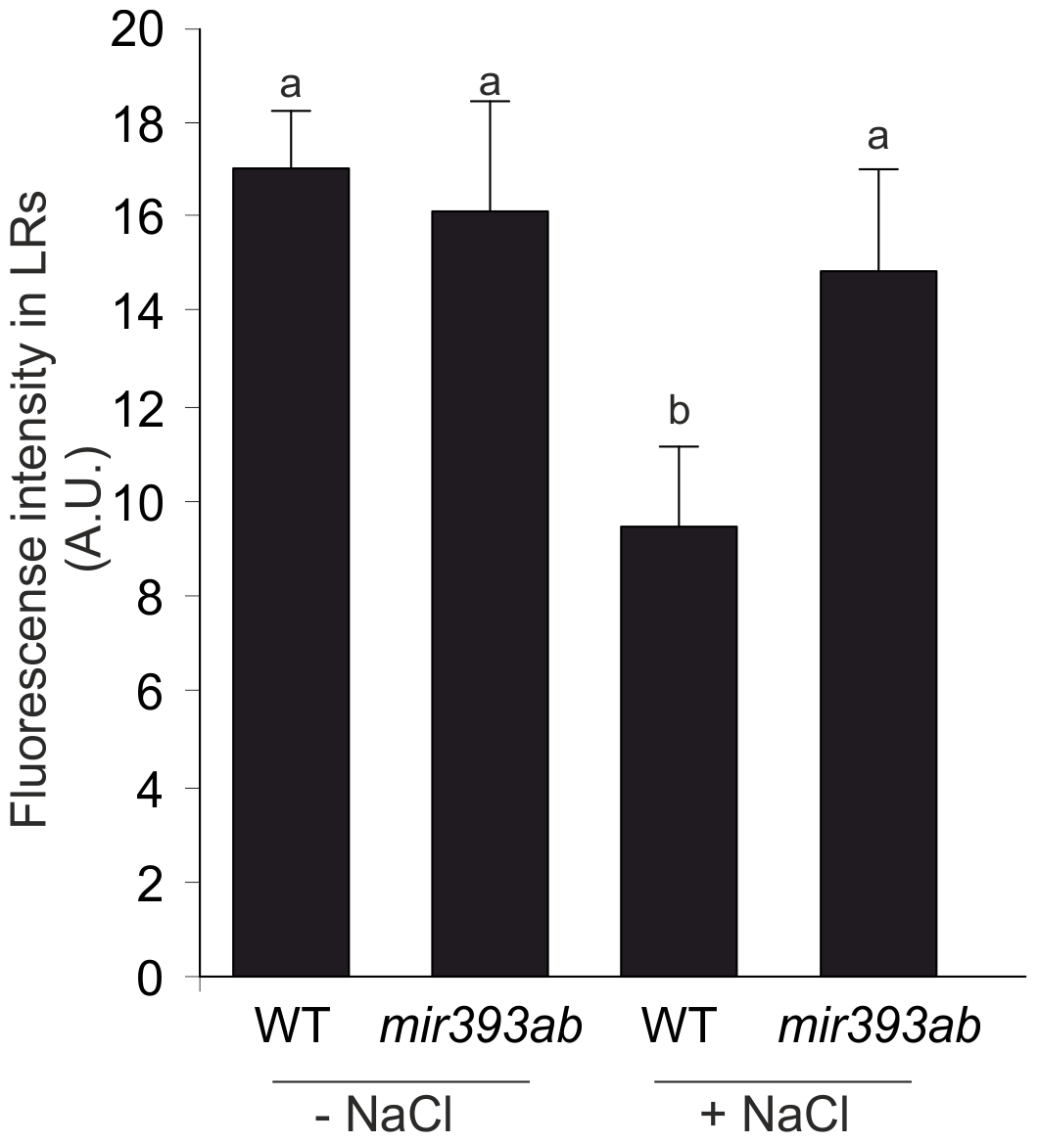
ROS accumulation in LR of *mir393ab* seedlings during acclimation to salinity. Four dpg WT and *mir393ab* seedlings were transferred onto ATS medium containing 75 mM NaCl. After 5 d of treatment, endogenous H_2_O_2_ was detected *in situ* by H2DCF DA probe. Quantification of probe fluorescence intensity in LRs is shown. LR were selected from the same position on the primary root. a.u., arbitrary units. Data are mean values (±SE) of three independent experiments. Different letters indicate a significant difference at P≤0.05 (Tukey test).

According to previous findings, an interlink between auxin and ROS was proposed to regulate growth and plant defense in responses to stress [17]. However, the precise mechanism remains to be elucidated. Hence, we focused on understanding how miR393-mediated repression of TIR1 influences ROS accumulation and antioxidant components during salinity. First, we analyzed endogenous ROS levels *in situ* in LRs of *mir393ab* and WT seedlings after 5 d of 75 mM NaCl treatment by using H2DCF DA probe. *mir393ab* seedlings showed 2-fold higher level of ROS in LRs under 75 mM NaCl (Figure 5). However, in WT plants, where auxin signaling is down-regulated, inhibition of LR development was associated to a concomitant reduction of ROS levels.

**Figure 6:**
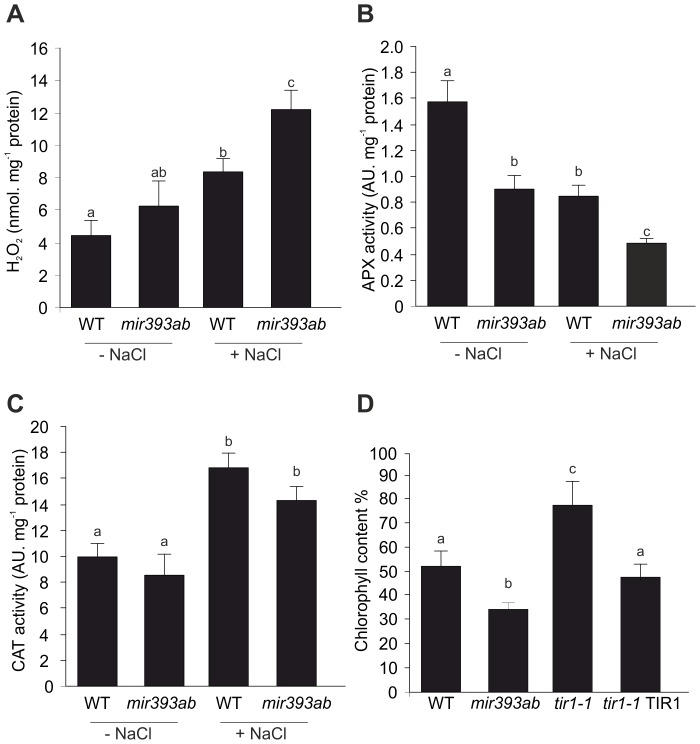
miR393 early regulates redox components under salt. Seven dpg WT and *mir393ab* seedlings were transferred onto liquid ATS medium supplemented with 100 mM NaCl. After 12 h of initial treatment (**A**) H_2_O_2_ accumulation, (**B**) APX activity and (**C**) CAT activity were measured. (**D**) Seven dpg *mir393ab*, *tir1-1*, *tir1-1 35Spro:TIR-Myc* and WT seedlings were treated with 100 mM NaCl for 3 d. Chlorophyll content was measured and expressed as percentage of untreated seedlings. Data are mean values (±SE) of three independent experiments. Different letters indicate a significant difference at P≤0.05 (Tukey test).

In a previous work, we reported that *tir1 afb2* mutant with reduced auxin response exhibits reduced levels of ROS under salinity compared to WT seedlings [18]. We then hypothesized that repression of auxin signaling through miR393 action could reduce the ROS burst that is generated by salt stress with detrimental effects on cellular processes.To further explore miR393 action on auxin regulation of ROS homeostasis, H_2_O_2_ was measured in seedlings treated with 100 mM NaCl for 12 h when an induction of H_2_O_2_ levels in WT plants was previously described [18], [47]. However, compared with WT, *mir393ab* seedlings showed an increase of more than 50% in peroxide accumulation after salt treatment while a slight increase was observed under standard conditions (Figure 6A). O_2_^−.^ content in leaves of NaCl-treated plants was also higher in *mir393ab* mutant compared with WT, as evidenced by in situ O_2_ - detection through NBT assay (Figure S10). In order to alleviate deleterious effects of ROS, plants employ defence systems that include non-enzymatic antioxidant compounds such as AA and glutathione and ROS scavenging enzymes. We hypothesized that the increased levels of ROS in *mir393ab* mutant plants under stress could be explained by a repression of the antioxidant metabolism. Consistent with this idea, a 56% reduction of APX enzymatic activity was observed in *mir393ab* compared with WT seedlings either in absence or presence of NaCl (Figure 6B). Catalase enzymatic activity was also measured but no difference was detected between *mir393ab* and WT seedlings (Figure 6C), probably indicating a specificity in the antioxidant enzyme regulation mediated by miR393 during salinity. Antioxidant metabolites, AA and GSH did not show significant changes between *mir393ab* and WT seedlings under either standard or salt-conditions (Table S1) while both of them were slightly reduced in *mir393ab* seedlings.

Finally, on the basis of the strong and rapid inhibitory effect of NaCl on auxin responses as well as the likely role of miR393 regulation on auxin signaling and ROS metabolism during salinity, we speculated that the repression of the auxin pathway is an important aspect of the defence response. Loss of chlorophyll is one of the most evident symptoms during oxidation by salt stress. Therefore, 7 dpg seedlings were transferred from solid ATS medium to liquid ATS medium containing 100 mM NaCl and after 3 d of salt treatment, the chlorophyll level was quantified in mutants and WT seedlings. As shown in Figure 6D, *mir393ab* showed a clear reduction of chlorophyll content under salt treatment compared with WT plants, suggesting enhanced damage under salinity. Conversely, *tir1-1* conserves 30% more chlorophyll than the WT upon salinity. Overexpression of TIR1 protein in the *tir1-1* background rescued the WT sensitivity to NaCl.

## Discussion

High salt concentration in productive soil arrests the plant's ability to take up water and grow. Thus, understanding the strategies that plants evolved to cope with salinity is of agronomic importance. Our study reveals how Arabidopsis seedlings regulate the auxin signaling pathway in order to modulate auxin-mediated morphological responses during NaCl-mediated salinity. In this work, we demonstrate that salinity induces the expression of miR393, primarily by enhancing the expression of *MIR393A*. In turn, miR393 negatively regulates TIR1 and AFB2 mRNA to lead to the stabilization of Aux/IAA repressors and to concomitant repression of auxin signaling. In addition, we report how miR393-mediated auxin down-regulation can influence root architecture during acclimation to salinity.

Depending on the type, intensity and duration of environmental stimuli as well as the plant developmental stage, auxin regulation could be controlled at different levels. For example, changes in auxin homeostasis and redistribution have been reported for plants growing under osmotic or mild salt stresses [2], [48], [49], [50]. When other ways by which NaCl might inhibit auxin response to salinity were explored, no difference in the concentration of free IAA was detected in WT seedlings under salt treatment (data not shown). Although we cannot rule out that auxin biosynthesis or transport could be also affected by NaCl at specific tissues or times, we assert that auxin signaling would be a dominant level of auxin regulation for acclimation during salinity. Since TIR1 and AFB2 are down-regulated during salt stress, we suggest that these two auxin receptors could be functionally required. However, while AFB2 showed a dose response to NaCl from 50 to 200 mM NaCl, TIR1 showed a similar repression from 50 mM NaCl to higher concentrations. TIR1 and AFB2 have been described as the dominant auxin receptors during seedling root growth, and biochemical differences between members of the auxin receptor family were detected and associated with the complexity of auxin response [9].

In order to get insights into the mode by which auxin signaling is repressed during acclimation to stress, the posttranscriptional regulation of auxin receptors by miR393 was further investigated. Previously, it has been reported that the locus-specific control of miR393 transcription can provide an additional layer of regulation for the auxin-signaling network through repressing target gene expression [15]. For instance, repression of auxin signaling through *AtMIR393A* action has been described in biotic stress response to *Pseudomonas* in Arabidopsis [13]. In addition, *AtMIR393B* has been reported to be the most important miR393 precursor involved in auxin-related development of leaves [11], [15]. In this current work, we showed that the promoter of *AtMIR393A* is activated by salt in whole seedlings, including shoots, principal root and LR. GUS induction in *MIR393Apro:GUS* lines during salt stress correlated with the repression of GUS activity in the *TIR1pro:TIR1-GUS* and *AFB2pro:AFB2-GUS* lines. These complementary patterns of *MIR393pro:GUS* and TIR1/AFB2 expression have been demonstrated during normal development of roots [9].

Salt treatment was unable to induce AXR3-GUS stability and to repress auxin response when *HSpro:AXR3-GUS* and *DR5pro:GUS* were analyzed in the *mir393ab* background (Figure S6). In addition to these facts, TIR1 level was not affected by salt in *mir393ab* seedlings and salt treatment did not reduce GUS staining in the resistant *TIR1pro:mTIR-GUS* line. Based on these observations, we propose that miR393-mediated posttranscriptional regulation of auxin receptors might be a crucial component of plant acclimation to salinity. Single mutant, *mir393a* and *mir393b* were not impaired in the down-regulation of TIR1 during salt stress (Figure S11A). We suspect that this is due to the fact that they are not null mutants, and therefore still accumulate sufficiently high levels of miR393 [26]. Such behavior of single mutants with a slight effect on *mir393a* mutant was also observed in biochemical and physiological responses including chlorophyll levels and LR number after salt exposition (Figure S11, B and C, respectively). Quantification of miR393 in roots by Northern blot assay indicated that miR393 is effectively induced (approximately 20%) in NaCl-treated seedlings (Figure S7). Importantly, although an induction was also detected in *mir393ab* mutant during salinity its level was more than 50% lower than in WT plants. We detected a slight reduction of miR393 levels after 1 h of salt treatment. However, we do not know whether this decrease has a biological significance for response to salt stress or whether this could suggest that other unidentified mechanisms contribute to the complex homeostasis of TIR1 and AFB2 regulation during acclimation to salinity.

Plants exposed to mild abiotic stress conditions exhibit different kind of stress-induced morphogenic responses (SIMR). SIMR has been postulated as part of a plant general acclimation strategy, whereby growth is reprogrammed to reduce exposure to stress [51], [52]. Frequently, observed symptoms in plant adaptive responses to salinity include growth retardation. In this direction, high salinity was reported to inhibit PR and LR growth [53], [54]. However, the adjustment of root growth to salinity seems to be less clear compared with other abiotic stresses [55]. Here, we explored the function of miR393-mediated modulation of auxin signaling in regulating root growth to reveal one putative mechanism by which salt could control root system architecture. Given the importance of root architecture during stress and the fact that, each organ might have different response programs during acclimation to stress we focused on the analysis of LR. Consistent with this fact, WT and *mir393ab* showed a reduction in the LR number during salinity but the amplitude of this reduction was much lower in *mir393ab* seedlings, suggesting an inability of this mutant to redirect root growth and development under salinity. Genetic and physiological evidence suggests that auxin is required at several specific developmental stages to facilitate LR formation [45]. A more precise analysis of the pattern of LR development in the *mir393ab* mutant suggested that miR393 mediates the inhibition of LR initiation and elongation when plants grow under salinity. Previous studies have postulated that changes in auxin levels by treatment with the auxin-transport inhibitor naphthylphthalamic acid (NPA) decreased the number and density of LR in *A. thaliana* plants [56].

Parry et al. [9] reported the expression of miR393 along the central stele in the primary root and later stages of LR development. Nevertheless, when seedlings were exposed to 200 mM NaCl for 2 h an activation of *MIR393A* promoter was detected in emergent and mature LR. Cross-sectional analysis of *MIR393Apro:GUS* roots showed that salinity induces *MIR393A* promoter activity in pericycle cells, which are stimulated to differentiate and proliferate to form primordia RL [31]. It was demonstrated that the local auxin accumulation in root pericycle cells is a specific and sufficient signal to specify pericycle cells into LRs founder cells [57]. Therefore, induction of miR393 in the pericycle cells with the consequent suppression of auxin signaling mediated by TIR1/AFBs could be an effective mechanism for cell-specific regulation of LR organogenesis during salt stress. Recently, it was demonstrated that endodermis is a tissue-specific cell layer where abscisic acid (ABA) signaling acts to regulate LR growth under salt-stress conditions [46]. According to Geng et al. [58] and to our results, a dynamic regulation of multiple hormonal signaling pathways involving auxin, ABA, gibberellic acid (GA), jasmonic acid and brassinosteroids should be necessary for temporal regulation of root patterning during acclimation to salinity.

In addition, *mir393ab* mutant failed in NaCl-mediated inhibition of PR elongation and rosette growth suggesting that miR393 is involved in different SIMR during salinity (Figure 4E and Figure S9).

Our findings are also consistent with results obtained in other systems where miR393 overexpression by stress has been reported. For instance, the overexpression of Arabidopsis *AtMIR393A* gene in tobacco modified auxin response and enhanced tolerance to salt stress [59]. Even more, miR393 up-regulation has been also described for other abiotic stresses such as cold, dehydration, and metal toxicity [41], [60], [61] but so far, the role of miR393 in these responses has not been explored.

Again in relation to SIMR, ROS and auxin signaling have been pointed out as important players in the regulatory networks that operate during adaptation to stress [17]. The mechanisms underlying the crosstalk between auxin and ROS and its impact on growth regulation remains to be elucidated. It is known that under various adverse environmental conditions, ROS homeostasis can lead to oxidative damage and cell death [62]. However, a multifaceted network of ROS producing and ROS-scavenging enzymes define a key homeostasis, from which ROS are capable to act as signals in different cellular processes [63]. Hence, ROS can result in potent signaling molecules that adjust growth, development and plant defense mechanism to stress [64]. In addition, an interaction between auxin and ROS signaling has been suggested during salinity by using *tir1 afb2* mutant [18]. Compared with WT, *tir1 afb2* plants showed significantly reduced ROS accumulation, higher antioxidant enzymatic activities as well as increased levels of AA revealing that down-regulation of auxin signaling impacts ROS metabolism under salinity. In order to provide new insights into the mechanism by which auxin and ROS could be regulated in plants growing under salt stress conditions, *mir393ab* seedlings were analyzed. Coinciding with the altered root architecture, an enhanced endogenous accumulation of ROS was showed in LR of *mir393ab* seedlings after 5 d of NaCl treatment. In WT plants, where auxin signaling is down-regulated by salinity, we detected an inhibition of LR development with a concomitant reduction of ROS levels. It has been recently described that auxin-mediated LR formation involved H_2_O_2_ generation [65]. Furthermore, exogenous H_2_O_2_ treatments mimics LR induction mediated by auxin [66] and H_2_O_2_ is also required for auxin-induced adventitious root formation in mung bean [67], [68]. Auxin also induces ROS production in maize developmental processes such as cell elongation of hypocotyls and the phenomenon of gravitropism [69], [70], [71]. Recent evidence proposed that auxin induces ROS production through the modulation of the NAD(P)H oxidase RbohD activity [72]. In this work, we found that *mir393ab* failed to counteract ROS accumulation evidenced by higher levels of ROS in roots as well as a reduction of APX enzymatic activity after 12 h of NaCl treatment, suggesting that auxin signaling could induce ROS through repression of the antioxidant system. Auxin negatively regulates the expression of *APX1* and *Zat12* transcription factor, which in turn regulates the expression of *APX1* [18]. In addition, Correa-Aragunde et al. [73] demonstrated that APX1 activity is inhibited by auxin-mediated denitrosylation. The current findings that the *mir393*-deficient mutant exhibits changes in APX but not in other antioxidant compounds such as AA and GSH, allowed us to suggest that specific components of redox control are subject to miR393-mediated auxin signaling regulation.

The plant antioxidant system consists of a number of enzymes and antioxidant compounds and this network was reported to be important for controlling excessive ROS production. However, the status of the antioxidant system is the result of changes in specific antioxidants depending on the type of stress, organ, tissue, cell and timing of the plant developmental program [63]. For instance, Barth et al. [74] reported that ascorbate deficient Arabidopsis mutant *vct1-1* is effective in counteracting ROS during pathogen infection and suggested that the low intracellular level of ascorbate could be sufficient for ROS scavenging. APX activity represents a key component of the AA-GSH cycle involved in the major antioxidant system of plant cells contributing to cellular ROS homeostasis [75]. The disruption of APX activity (without changes in AA and GSH) might lead to increased steady state levels of oxidants in *mir393ab* cells affecting the root system. It was already reported that cytosolic APX1 knock-out plants present higher levels of H_2_O_2_ and oxidative damage, showing growth retardation especially under stress conditions [76], [77]. Recently, it was reported that PR elongation and LR formation is altered in response to auxin in the *apx1* mutant [73]. Their data indicate that auxin treatment induces H_2_O_2_ accumulation in Arabidopsis roots through auxin-mediated partial denitrosylation of APX1. Furthermore, exogenous H_2_O_2_ treatments results in inhibition of PR elongation and induction of LR formation, a phenotype reminiscent to the phenotype found in *mir393ab* seedlings and auxin-treated roots ([66]; this work). According to these, APX1 regulation exerted by miR393 may be a specific mechanism involved in the appropriate redistribution of H_2_O_2_ accumulation during root growth and LR development in Arabidopsis.

**Figure 7:**
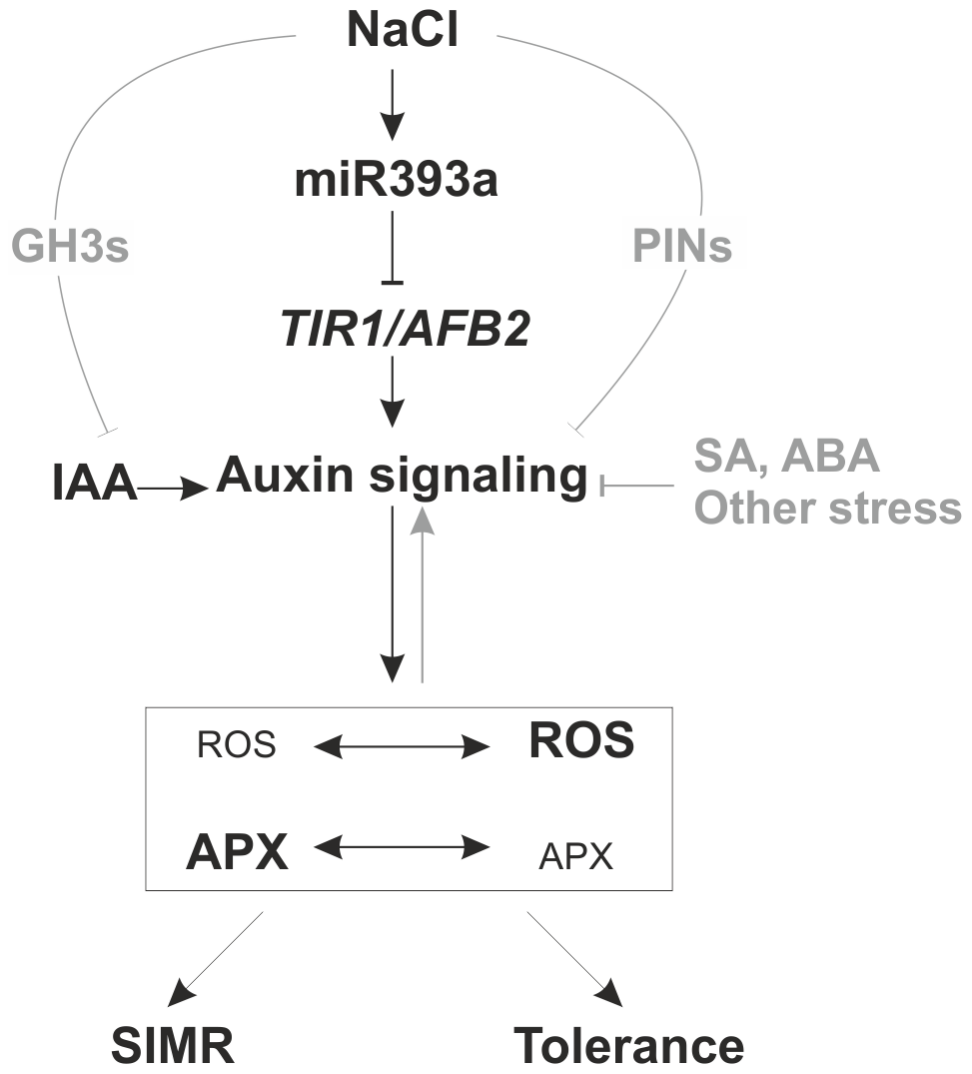
Modulation of plant development and acclimation to salinity are interconnected responses influenced by miR393 regulation node. These two processes are strongly regulated by reciprocal interaction between ROS and auxin signaling pathway. NaCl-mediated stress would conduct to miR393 induction that targets TIR1-AFB2 auxin receptors leading to stabilization of Aux/IAA repressors and down- regulation of auxin signaling pathway. In turn, auxin modulation influences on ROS-associated metabolism. Salt exposition leading to SIMR would be under negative regulation of the auxin signaling pathway. Depending on time and intensity of salt stress, early ROS-auxin crosstalk may exert impact on SIMR and tolerance to salinity. Others hormones and/or miRNAs may also regulate auxin-dependent pathway and physiological responses during acclimation to salinity.

Finally, a putative mechanistic model that may take place during SIMR in order to develop tolerance to salinity was described. An integrative miR393 post-transcriptional down-regulation of auxin signaling may be a regulatory module by which plants redirect plant growth and development through the modulation of ROS-associated metabolism in order to reallocate metabolic resources to defense responses and acclimation (Figure 7). Then, depending on the environmental stimuli a general acclimation strategy could help to compensate the stress-mediated redox imbalance and growth signals to control the reprogramming of plant development under stress. Lastly, it would be interesting to determine the endogenous sources of ROS as well as the downstream consequences of ROS regulation in stressed tissues. In addition, Blomster et al. [22] reported that apoplastic ROS mediated by O_3_ modified several aspects of auxin homeostasis and signaling. These authors also postulated that ROS could suppress the auxin pathway by decreasing TIR/AFBs expression independently of miR393 and SA. In conclusion, future studies will be important to identify additional convergence points between ROS and auxin signaling and to explore specific methods to precisely quantify ROS to give deeper evidence on miR393-mediated regulation of ROS metabolism.

## Supporting Information

Figure S1 **Salinity effect on 2,4-D-mediated LR development.** Four dpg WT seedlings were transferred from auxin-free medium onto ATS medium containing no auxin or 85 nM 2,4-D in combination with increasing concentrations of NaCl. The total number of emerged lateral roots was counted 4 d after the transfer to new media. Data are mean values (±SE) of three independent experiments. Different letters indicate a significant difference at P≤0.05 (Tukey test).
(TIF)

Figure S2 **Salinity effect on** ***MIR393Apro:GUS*** **,** ***MIR393Bpro:GUS*** **and** ***TIR1pro:TIR1-GUS*** **seedlings.** Seven dpg (**A**) *MIR393Apro:GUS*, (**B**) *MIR393Bpro:GUS* and (**C**) *TIR1pro:TIR1-GUS* seedlings were transferred to liquid ATS medium supplemented with 200 mM NaCl for designated times. Representative photographs of root tips and the GUS signal detected in NaCl treatment relative to control is shown. The control value is arbitrarily set to 1 in each case.
(TIF)

Figure S3 **AFB2 relative transcript levels in** ***ago1-27*** **and WT seedlings upon NaCl treatments.** Seven dpg WT and *ago1-27* seedlings were subjected to 200 mM NaCl treatment for 4 h. Relative transcript level of AFB2 upon treatment was measured by RT-PCR. The control value is arbitrarily set to 1 in each case. Data are mean values (±SE) of three independent experiments. Different letters indicate a significant difference at P≤0.05 (Tukey test).
(TIF)

Figure S4 ***MIR393A*** **promoter activity in cross sections of** ***MIR393A::GUS*** **roots upon NaCl.** Seven dpg *MIR393Apro:GUS* seedlings were transferred to liquid ATS medium supplemented with 200 mM NaCl for 2 h. Seedlings were included in a paraffin matrix (Paraplast) at 60°C and roots were cut into 5 µm sections using a Minot type rotary microtome Zeiss HYRAX M 15. Section were deparaffined with xylene, mounted with Entellan and observed by bright field microscopy in an Olympus CX21 microscope. Images were captured using a digital camera attached to the microscope. e: endodermis; p: pericycle; Cb: Casparian band; x: xylem. The control value of GUS staining is arbitrarily set to 1. Data are mean values of 3 independent experiments (n = 6).
(TIF)

Figure S5 ***MIR393B*** **promoter activity upon NaCl treatment in** ***AtMIR393Bpro:GUS*** **plants.** (**A**) Seven dpg *AtMIR393Bpro:GUS* seedlings were transferred to liquid ATS medium supplemented with increasing concentrations of NaCl for 2 h. GUS activity was revealed after incubation with X-Gluc at 37°C. GUS staining in representative leaves and root segments are shown. (**B**) Relative transcript level of GUS upon 200 mM NaCl treatment as described in (A). The control value is arbitrarily set to 1 in each case. Data are mean values (±SE) of three independent experiments.
(TIF)

Figure S6 **Salinity effect on** ***HSpro:AXR3NT-GUS*** **and** ***DR5pro:GUS*** **in** ***mir393ab*** **background.** Seven dpg seedlings were incubated in liquid ATS medium with increasing concentrations of NaCl for 4 h and then subjected to GUS staining. Representative photographs of root tips of *mir393ab HSpro:AXR3NT-GUS* (A) and *mir393ab DR5pro:GUS* (B). In (A) seedlings were previously treated for 2 h at 37°C. The control value is arbitrarily set to 1 in each case. Data are mean values of three independent experiments.
(TIF)

Figure S7 **miR393 levels during salinity in roots.** Small RNA blot hybridization of RNA (25 µg) from roots and shoots of 7 dpg seedlings treated with 200 mM NaCl for designated times. Probed sRNAs are indicated on the right. The signal detected in mutants relative to control is normalized to signals for the unrelated miR171. The control value is arbitrarily set to 1 in each case.
(TIF)

Figure S8 ***tir1 afb2*** **and** ***mir393ab*** **root morphological responses.** Four dpg WT, *mir393ab* and *tir1 afb2* seedlings were transferred onto ATS medium containing 75 mM NaCl. Representative photographs of *tir1afb2* seedlings after 5 d of treatment are shown in (**A**). LRs were quantified at designed times (**B**). (**C**) PR length of WT, *mir393ab* and *tir1afb2* seedlings was measured after 5 d of treatment. Data are mean values (±SE) of three independent experiments.
(TIF)

Figure S9 ***mir393ab*** **morphological response in leaves.** WT and *mir393ab* seedlings were grown in ATS medium supplemented with or without 75 mM NaCl in horizontal position. Rosette area was measured after 12 d of treatment by finding the minimal circle area that contained all leaves. Data are mean values (±SE) of three independent experiments. Different letters indicate a significant difference at P≤0.05 (Tukey test).
(TIF)

Figure S10 O_2_^−.^ level in *mir393ab* mutant under salinity. Fourteen dpg WT and *mir393ab* leaves were transferred onto liquid ATS medium supplemented with 100 mM NaCl. After 12 h of initial treatment in situ O_2_^−.^ accumulation was detected by NBT staining. Representative photographs are shown.
(TIF)

Figure S11 Analysis of single mutants *mir393a* and *mir393b*. (**A**) Seven dpg seedlings were subjected to 200 mM NaCl treatment for 4 h. Relative transcript level of TIR1 upon treatment was measured by RT-PCR. The control value is arbitrarily set to 1 in each case. Data are mean values (±SE) of three independent experiments. (**B**) Four dpg seedlings were transferred onto ATS medium containing 75 mM NaCl. LR were quantified after 5 d of treatment. Data are mean values (±SE) of three independent experiments. (**C**) Seven dpg seedlings were treated with 100 mM NaCl for 3 d. Chlorophyll content was measured and expressed as percentage of untreated seedlings. Data are mean values (±SE) of three independent experiments. Different letters indicate a significant difference at P≤0.05 (Tukey test).
(TIF)

Table S1 **Antioxidant levels in WT and** ***mir393ab*** **plants under salinity.** Seven dpg WT and *mir393ab* seedlings were subjected to 100 mM NaCl treatment. After 12 h, AA and GSH levels were quantified. Data are mean values (±SE) of three independent experiments. Different letters indicate significant difference at P≤0.05 (Tukey test).
(TIF)

## References

[1] Takeda S, Matsuoka M (2008) Genetic approaches to crop improvement: Responding to environmental and population changes. Nat Rev Genet 9: 444–457. 18475268 10.1038/nrg2342

[2] Park JE, Park JY, Kim YS, Staswick PE, Jeon J, et al (2007) GH3-mediated auxin homeostasis links growth regulation with stress adaptation response in Arabidopsis. J of Biol Chem 282: 10036–10046. 17276977 10.1074/jbc.M610524200

[3] Wolters H, Jurgens G (2009) Survival of the flexible: hormonal growth control and adaptation in plant development. Nat Rev Genet 10: 305–317. 19360022 10.1038/nrg2558

[4] Gray WM, Kepinski S, Rouse D, Leyser O, Estelle M (2001) Auxin regulates SCF(TIR1)-dependent degradation of AUX/IAA proteins. Nature 414: 271–276. 11713520 10.1038/35104500

[5] Dharmasiri N, Dharmasiri S, Estelle M (2005) The F-box protein TIR1 is an auxin receptor. Nature 435: 441–445. 15917797 10.1038/nature03543

[6] Dharmasiri N, Dharmasiri S, Weijers D, Lechner E, Yamada M, et al (2005) Plant development is regulated by a family of auxin receptor F box proteins. Dev Cell 9: 109–119. 15992545 10.1016/j.devcel.2005.05.014

[7] Kepinski S, Leyser O (2005) The Arabidopsis F-box protein TIR1 is an auxin receptor. Nature 435: 446–451. 15917798 10.1038/nature03542

[8] Hagen G, Guilfoyle T (2002) Auxin-responsive gene expression: genes, promoters and regulatory factors. Plant Mol Biol 49: 373–385. 12036261

[9] Parry G, Calderon-Villalobos LI, Prigge M, Peret B, Dharmasiri S, et al (2009) Complex regulation of the TIR1/AFB family of auxin receptors. Proc Natl Acad Sci U S A 106: 22540–22545. 20018756 10.1073/pnas.0911967106 PMC2799741

[10] Calderon Villalobos LI, Lee S, De Oliveira C, Ivetac A, Brandt W, et al (2012) A combinatorial TIR1/AFB-Aux/IAA co-receptor system for differential sensing of auxin. Nat Chem Biol 8: 477–485. 22466420 10.1038/nchembio.926 PMC3331960

[11] Chen ZH, Bao ML, Sun YZ, Yang YJ, Xu XH, et al (2011) Regulation of auxin response by miR393-targeted transport inhibitor response protein 1 is involved in normal development in Arabidopsis. Plant Mol Biol 77: 619–629. 22042293 10.1007/s11103-011-9838-1

[12] Windels D, Vazquez F (2011) miR393: integrator of environmental cues in auxin signaling? Plant Signal Behav 6: 1672–1675. 22067993 10.4161/psb.6.11.17900 PMC3329333

[13] Navarro L, Dunoyer P, Jay F, Arnold B, Dharmasiri N, et al (2006) A plant miRNA contributes to antibacterial resistance by repressing auxin signaling. Science 312: 436–439. 16627744 10.1126/science.1126088

[14] Vidal EA, Araus V, Lu C, Parry G, Green PJ, et al (2010) Nitrate-responsive miR393/AFB3 regulatory module controls root system architecture in Arabidopsis thaliana. Proc Natl Acad Sci U S A 107: 4477–4482. 20142497 10.1073/pnas.0909571107 PMC2840086

[15] Si-Ammour A, Windels D, Arn-Bouldoires E, Kutter C, Ailhas J, et al (2011) miR393 and secondary siRNAs regulate expression of the TIR1/AFB2 auxin receptor clade and auxin-related development of Arabidopsis leaves. Plant Physiol 157: 683–691. 21828251 10.1104/pp.111.180083 PMC3192580

[16] Miller G, Suzuki N, Ciftci-Yilmaz S, Mittler R (2010) Reactive oxygen species homeostasis and signalling during drought and salinity stresses. Plant Cell Environ 33: 453–467. 19712065 10.1111/j.1365-3040.2009.02041.x

[17] Tognetti VB, Muhlenbock P, Van Breusegem F (2012) Stress homeostasis - the redox and auxin perspective. Plant Cell Environ 35: 321–333. 21443606 10.1111/j.1365-3040.2011.02324.x

[18] Iglesias M, Terrile M, Bartoli C, D'Ippólito S, Casalongué C (2010) Auxin signaling participates in the adaptative response against oxidative stress and salinity by interacting with redox metabolism in Arabidopsis. Plant Mol Biol 74: 215–222. 20661628 10.1007/s11103-010-9667-7

[19] Cheong YH, Chang HS, Gupta R, Wang X, Zhu T, et al (2002) Transcriptional profiling reveals novel interactions between wounding, pathogen, abiotic stress, and hormonal responses in Arabidopsis. Plant Physiol 129: 661–677. 12068110 10.1104/pp.002857 PMC161692

[20] Jain M, Khurana JP (2009) Transcript profiling reveals diverse roles of auxin-responsive genes during reproductive development and abiotic stress in rice. Febs J 276: 3148–3162. 19490115 10.1111/j.1742-4658.2009.07033.x

[21] Van Hoewyk D, Takahashi H, Inoue E, Hess A, Tamaoki M, et al (2008) Transcriptome analyses give insights into selenium-stress responses and selenium tolerance mechanisms in Arabidopsis. Physiol Plant 132: 236–253. 18251864 10.1111/j.1399-3054.2007.01002.x

[22] Blomster T, Salojarvi J, Sipari N, Brosche M, Ahlfors R, et al (2011) Apoplastic reactive oxygen species transiently decrease auxin signaling and cause stress-induced morphogenic response in Arabidopsis. Plant Physiol 157: 1866–1883. 22007024 10.1104/pp.111.181883 PMC3327221

[23] Ruegger M, Dewey E, Gray WM, Hobbie L, Turner J, et al (1998) The TIR1 protein of Arabidopsis functions in auxin response and is related to human SKP2 and yeast grr1p. Genes Dev 12: 198–207. 9436980 10.1101/gad.12.2.198 PMC316440

[24] Savaldi-Goldstein S, Baiga TJ, Pojer F, Dabi T, Butterfield C, et al (2008) New auxin analogs with growth-promoting effects in intact plants reveal a chemical strategy to improve hormone delivery. Proc Natl Acad Sci U S A 105: 15190–15195. 18818305 10.1073/pnas.0806324105 PMC2567513

[25] Morel JB, Godon C, Mourrain P, Beclin C, Boutet S, et al (2002) Fertile hypomorphic ARGONAUTE (ago1) mutants impaired in post-transcriptional gene silencing and virus resistance. Plant Cell 14: 629–639. 11910010 10.1105/tpc.010358 PMC150585

[26] Windels D, Bielewicz D, Ebneter M, Jarmolowski A, Szweykowska-Kulinska Z, et al (2014) miR393 Is Required for Production of Proper Auxin Signalling Outputs. PLoS One 9: e95972. 24763336 10.1371/journal.pone.0095972 PMC3999107

[27] Ulmasov T, Murfett J, Hagen G, Guilfoyle TJ (1997) Aux/IAA proteins repress expression of reporter genes containing natural and highly active synthetic auxin response elements. Plant Cell 9: 1963–1971. 9401121 10.1105/tpc.9.11.1963 PMC157050

[28] Oono Y, Chen QG, Overvoorde PJ, Kohler C, Theologis A (1998) age Mutants of Arabidopsis exhibit altered auxin-regulated gene expression. Plant Cell 10: 1649–1662. 9761792 10.1105/tpc.10.10.1649 PMC143942

[29] Terrile MC, Paris R, Calderon-Villalobos LI, Iglesias MJ, Lamattina L, et al (2012) Nitric oxide influences auxin signaling through S-nitrosylation of the Arabidopsis TRANSPORT INHIBITOR RESPONSE 1 auxin receptor. Plant J 70: 492–500. 22171938 10.1111/j.1365-313X.2011.04885.x PMC3324642

[30] Wilson AK, Pickett FB, Turner JC, Estelle M (1990) A dominant mutation in Arabidopsis confers resistance to auxin, ethylene and abscisic acid. Mol Gen Genet 222: 377–383. 2148800 10.1007/BF00633843

[31] Malamy JE, Benfey PN (1997) Organization and cell differentiation in lateral roots of Arabidopsis thaliana. Dev 124: 33–44. 10.1242/dev.124.1.33 9006065

[32] Rahman A, Bannigan A, Sulaman W, Pechter P, Blancaflor EB, et al (2007) Auxin, actin and growth of the Arabidopsis thaliana primary root. Plant J 50: 514–528. 17419848 10.1111/j.1365-313X.2007.03068.x

[33] Vazquez F, Blevins T, Ailhas J, Boller T, Meins F Jr (2008) Evolution of Arabidopsis MIR genes generates novel microRNA classes. Nucleic Acids Res 36: 6429–6438. 18842626 10.1093/nar/gkn670 PMC2582634

[34] Arnon DI (1949) Copper Enzymes in Isolated Chloroplasts. Polyphenoloxidase in Beta Vulgaris. Plant Physiol 24: 1–15. 16654194 10.1104/pp.24.1.1 PMC437905

[35] Jabs T, Dietrich RA, Dangl JL (1996) Initiation of runaway cell death in an Arabidopsis mutant by extracellular superoxide. Science 273: 1853–1856. 8791589 10.1126/science.273.5283.1853

[36] Bellincampi D, Dipierro N, Salvi G, Cervone F, De Lorenzo G (2000) Extracellular H(2)O(2) induced by oligogalacturonides is not involved in the inhibition of the auxin-regulated rolB gene expression in tobacco leaf explants. Plant Physiol 122: 1379–1385. 10759534 10.1104/pp.122.4.1379 PMC58973

[37] Bradford M (1976) A rapid and sensitive method for the quantitation of micrograms quantities of protein utilizing the principle of protein-dye binding. Annal Biochem 72: 248–254. 10.1016/0003-2697(76)90527-3 942051

[38] Gergoff G, Chaves A, Bartoli CG (2010) Ethylene regulates ascorbic acid content during dark-induced leaf senescence. Plant Sci 178: 207–212.

[39] Griffith OW (1980) Determination of glutathione and glutathione disulfide using glutathione reductase and 2-vinylpyridine. Analytical Biochemistry 106: 207–212. 7416462 10.1016/0003-2697(80)90139-6

[40] Estelle M (2008) Phenotypic Analysis of Arabidopsis Mutants: Auxin Hormone Response. Cold Spring Harbor Protocols 2008: pdb.prot4965. 21356845 10.1101/pdb.prot4965

[41] Kruszka K, Pieczynski M, Windels D, Bielewicz D, Jarmolowski A, et al (2012) Role of microRNAs and other sRNAs of plants in their changing environments. J Plant Physiol 169: 1664–1672. 22647959 10.1016/j.jplph.2012.03.009

[42] Mallory A, Vaucheret H (2010) Form, function, and regulation of ARGONAUTE proteins. Plant Cell 22: 3879–3889. 21183704 10.1105/tpc.110.080671 PMC3027166

[43] Sunkar R, Zhu JK (2004) Novel and stress-regulated microRNAs and other small RNAs from Arabidopsis. Plant Cell 16: 2001–2019. 15258262 10.1105/tpc.104.022830 PMC519194

[44] Gustafson AM, Allen E, Givan S, Smith D, Carrington JC, et al (2005) ASRP: The Arabidopsis Small RNA Project Database. Nucleic Acids Research 33: D637–D640. 15608278 10.1093/nar/gki127 PMC540081

[45] Casimiro I, Beeckman T, Graham N, Bhalerao R, Zhang H, et al (2003) Dissecting Arabidopsis lateral root development. Trends Plant Sci 8: 165–171. 12711228 10.1016/S1360-1385(03)00051-7

[46] Duan L, Dietrich D, Ng CH, Chan PMY, Bhalerao R, et al (2013) Endodermal ABA Signaling Promotes Lateral Root Quiescence during Salt Stress in Arabidopsis Seedlings. Plant Cell 25: 324–341. 23341337 10.1105/tpc.112.107227 PMC3584545

[47] Guan Q, Wu J, Yue X, Zhang Y, Zhu J (2013) A Nuclear Calcium-Sensing Pathway Is Critical for Gene Regulation and Salt Stress Tolerance in Arabidopsis. PLoS Genet 9: e1003755. 24009530 10.1371/journal.pgen.1003755 PMC3757082

[48] Zhang SW, Li CH, Cao J, Zhang YC, Zhang SQ, et al (2009) Altered architecture and enhanced drought tolerance in rice via the down-regulation of indole-3-acetic acid by TLD1/OsGH3.13 activation. Plant Physiol 151: 1889–1901. 19776160 10.1104/pp.109.146803 PMC2785973

[49] Wang Y, Li K, Li X (2009) Auxin redistribution modulates plastic development of root system architecture under salt stress in Arabidopsis thaliana. J Plant Physiol 166: 1637–1645. 19457582 10.1016/j.jplph.2009.04.009

[50] Zhao Y, Wang T, Zhang W, Li X (2011) SOS3 mediates lateral root development under low salt stress through regulation of auxin redistribution and maxima in Arabidopsis. New Phytol 189: 1122–1134. 21087263 10.1111/j.1469-8137.2010.03545.x

[51] Potters G, Pasternak TP, Guisez Y, Palme KJ, Jansen MA (2007) Stress-induced morphogenic responses: growing out of trouble? Trends Plant Sci 12: 98–105. 17287141 10.1016/j.tplants.2007.01.004

[52] Potters G, Horemans N, Jansen MA (2010) The cellular redox state in plant stress biology–a charging concept. Plant Physiol Biochem 48: 292–300. 20137959 10.1016/j.plaphy.2009.12.007

[53] Burssens S, Himanen K, van de Cotte B, Beeckman T, Van Montagu M, et al (2000) Expression of cell cycle regulatory genes and morphological alterations in response to salt stress in Arabidopsis thaliana. Planta 211: 632–640. 11089675 10.1007/s004250000334

[54] He X-J, Mu R-L, Cao W-H, Zhang Z-G, Zhang J-S, et al (2005) AtNAC2, a transcription factor downstream of ethylene and auxin signaling pathways, is involved in salt stress response and lateral root development. Plant J 44: 903–916. 16359384 10.1111/j.1365-313X.2005.02575.x

[55] Zolla G, Heimer YM, Barak S (2010) Mild salinity stimulates a stress-induced morphogenic response in Arabidopsis thaliana roots. J Exp Bot 61: 211–224. 19783843 10.1093/jxb/erp290 PMC2791118

[56] Reed RC, Brady SR, Muday GK (1998) Inhibition of auxin movement from the shoot into the root inhibits lateral root development in arabidopsis. Plant Physiol 118: 1369–1378. 9847111 10.1104/pp.118.4.1369 PMC34753

[57] Dubrovsky JG, Sauer M, Napsucialy-Mendivil S, Ivanchenko MG, Friml J, et al (2008) Auxin acts as a local morphogenetic trigger to specify lateral root founder cells. Proc Natl Acad Sci U S A 105: 8790–8794. 18559858 10.1073/pnas.0712307105 PMC2438385

[58] Geng Y, Wu R, Wee CW, Xie F, Wei X, et al (2013) A Spatio-Temporal Understanding of Growth Regulation during the Salt Stress Response in Arabidopsis. Plant Cell 25: 2132–2154. 23898029 10.1105/tpc.113.112896 PMC3723617

[59] Feng X-M, You C-X, Qiao Y, Mao K, Hao Y-J (2010) Ectopic overexpression of Arabidopsis AtmiR393a gene changes auxin sensitivity and enhances salt resistance in tobacco. Acta Physiol Plant 32: 997–1003.

[60] Sunkar R, Chinnusamy V, Zhu J, Zhu JK (2007) Small RNAs as big players in plant abiotic stress responses and nutrient deprivation. Trends Plant Sci 12: 301–309. 17573231 10.1016/j.tplants.2007.05.001

[61] Mendoza-Soto AB, Sanchez F, Hernandez G (2012) MicroRNAs as regulators in plant metal toxicity response. Front Plant Sci 3: 105. 22661980 10.3389/fpls.2012.00105 PMC3356851

[62] Van Breusegem F, Dat JF (2006) Reactive Oxygen Species in Plant Cell Death. Plant Physiol 141: 384–390. 16760492 10.1104/pp.106.078295 PMC1475453

[63] Mittler R, Vanderauwera S, Gollery M, Van Breusegem F (2004) Reactive oxygen gene network of plants. Trends Plant Sci 9: 490–498. 15465684 10.1016/j.tplants.2004.08.009

[64] Apel K, Hirt H (2004) REACTIVE OXYGEN SPECIES: Metabolism, oxidative stress, and signal tranduction. Ann Rev Plant Biol 55: 373–379. 15377225 10.1146/annurev.arplant.55.031903.141701

[65] Ma F, Wang L, Li J, Samma M, Xie Y, et al (2013) Interaction between HY1 and H2O2 in auxin-induced lateral root formation in Arabidopsis. Plant Mol Biol 1–13. 10.1007/s11103-013-0168-3 24366686

[66] Wang P, Du Y, Li Y, Ren D, Song C-P (2010) Hydrogen Peroxide–Mediated Activation of MAP Kinase 6 Modulates Nitric Oxide Biosynthesis and Signal Transduction in Arabidopsis. Plant Cell 22: 2981–2998. 20870959 10.1105/tpc.109.072959 PMC2965546

[67] Li S-W, Xue L, Xu S, Feng H, An L (2009) IBA-induced changes in antioxidant enzymes during adventitious rooting in mung bean seedlings: The role of H2O2. Environ Exp Bot 66: 442–450.

[68] Bai M-Y, Fan M, Oh E, Wang Z-Y (2012) A Triple Helix-Loop-Helix/Basic Helix-Loop-Helix Cascade Controls Cell Elongation Downstream of Multiple Hormonal and Environmental Signaling Pathways in Arabidopsis. Plant Cell 24: 4917–4929. 23221598 10.1105/tpc.112.105163 PMC3556966

[69] Joo JH, Bae YS, Lee JS (2001) Role of Auxin-Induced Reactive Oxygen Species in Root Gravitropism. Plant Physiol 126: 1055–1060. 11457956 10.1104/pp.126.3.1055 PMC116462

[70] Schopfer P, Liszkay A, Bechtold M, Frahry G, Wagner A (2002) Evidence that hydroxyl radicals mediate auxin-induced extension growth. Planta 214: 821–828. 11941457 10.1007/s00425-001-0699-8

[71] Joo JH, Wang S, Chen JG, Jones AM, Fedoroff NV (2005) Different signaling and cell death roles of heterotrimeric G protein ά and β subunits in the Arabidopsis oxidative stress response to ozone. Plant Cell 17: 957–970. 15705948 10.1105/tpc.104.029603 PMC1069711

[72] Peer WA, Cheng Y, Murphy AS (2013) Evidence of oxidative attenuation of auxin signalling. J Exp Bot 64: 2629–2639. 23709674 10.1093/jxb/ert152

[73] Correa-Aragunde N, Foresi N, Delledonne M, Lamattina L (2013) Auxin induces redox regulation of ascorbate peroxidase activity by S-nitrosylation/denitrosylation balance resulting in changes of root growth pattern in Arabidopsis. J Exp Bot 64: 3339–3349. 23918967 10.1093/jxb/ert172

[74] Barth C, Moeder W, Klessig DF, Conklin PL (2004) The Timing of Senescence and Response to Pathogens Is Altered in the Ascorbate-Deficient Arabidopsis Mutant vitamin c-1. Plant Physiol 134: 1784–1792. 15064386 10.1104/pp.103.032185 PMC419851

[75] Foyer CH, Noctor G (2005) Redox homeostasis and antioxidant signaling: a metabolic interface between stress. Plant Cell 17: 1866–1875. 15987996 10.1105/tpc.105.033589 PMC1167537

[76] Davletova S, Rizhsky L, Liang H, Shengqiang Z, Oliver DJ, et al (2005) Cytosolic Ascorbate Peroxidase 1 Is a Central Component of the Reactive Oxygen Gene Network of Arabidopsis. Plant Cell 17: 268–281. 15608336 10.1105/tpc.104.026971 PMC544504

[77] Zhang Z, Zhang Q, Wu J, Zheng X, Zheng S, et al (2013) Gene Knockout Study Reveals That Cytosolic Ascorbate Peroxidase 2(OsAPX2) Plays a Critical Role in Growth and Reproduction in Rice under Drought, Salt and Cold Stresses. PLoS One 8: e57472. 23468992 10.1371/journal.pone.0057472 PMC3585366

