# Supplementary figures and images for: MiR393 Regulation of Auxin Signaling and Redox-Related Components during Acclimation to Salinity in Arabidopsis
Source: PLoS One. 2014 Sep 15;9(9):e107678. doi: 10.1371/journal.pone.0107678 (PMC4164656; doi:10.1371/journal.pone.0107678)

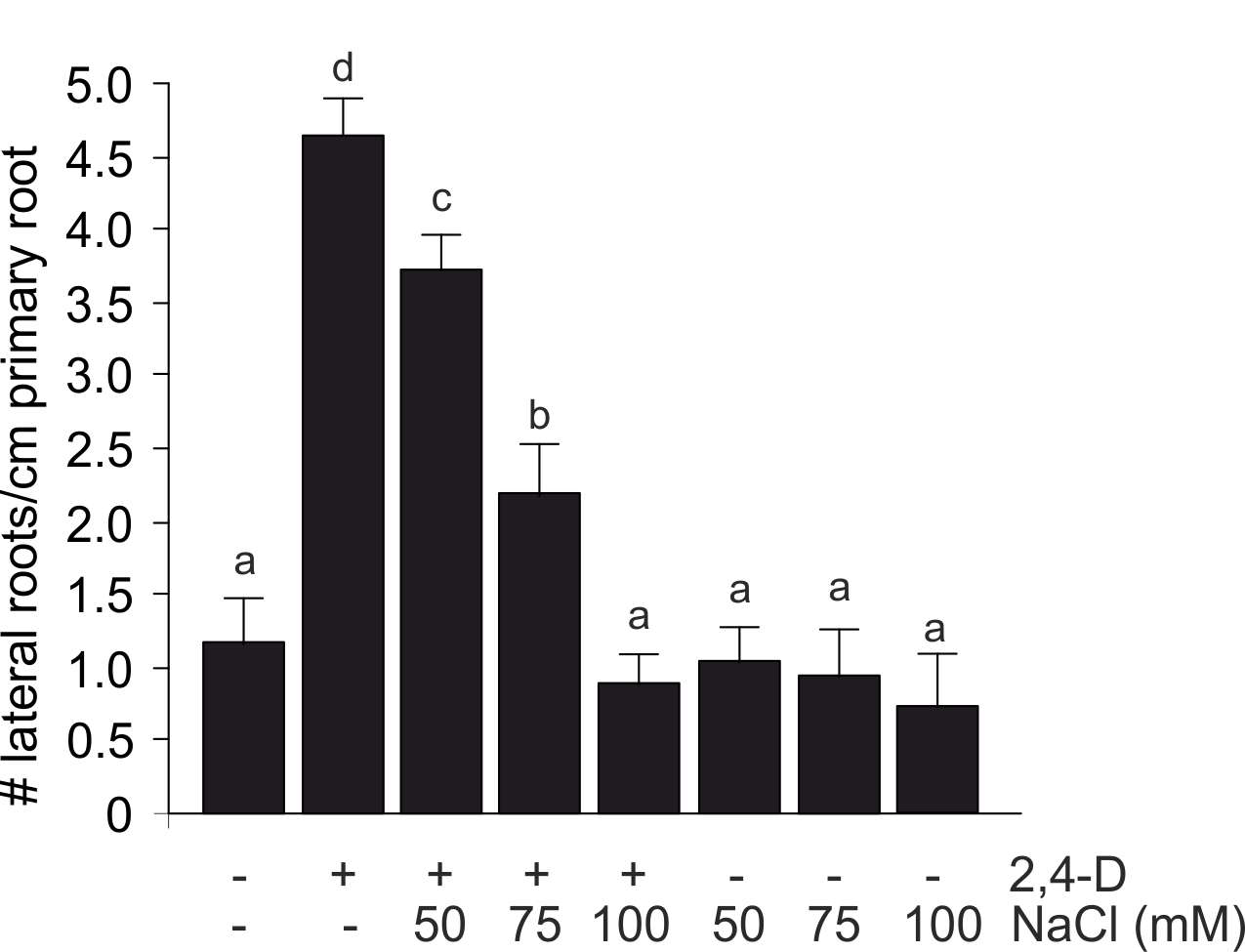

Supplement: Figure S1 — Salinity effect on 2,4-D-mediated LR development. Four dpg WT seedlings were transferred from auxin-free medium onto ATS medium containing no auxin or 85 nM 2,4-D in combination with increasing concentrations of NaCl. The total number of emerged lateral roots was counted 4 d after the transfer to new media. Data are mean values (±SE) of three independent experiments. Different letters indicate a significant difference at P≤0.05 (Tukey test). (TIF) [file pone.0107678.s001.tif]

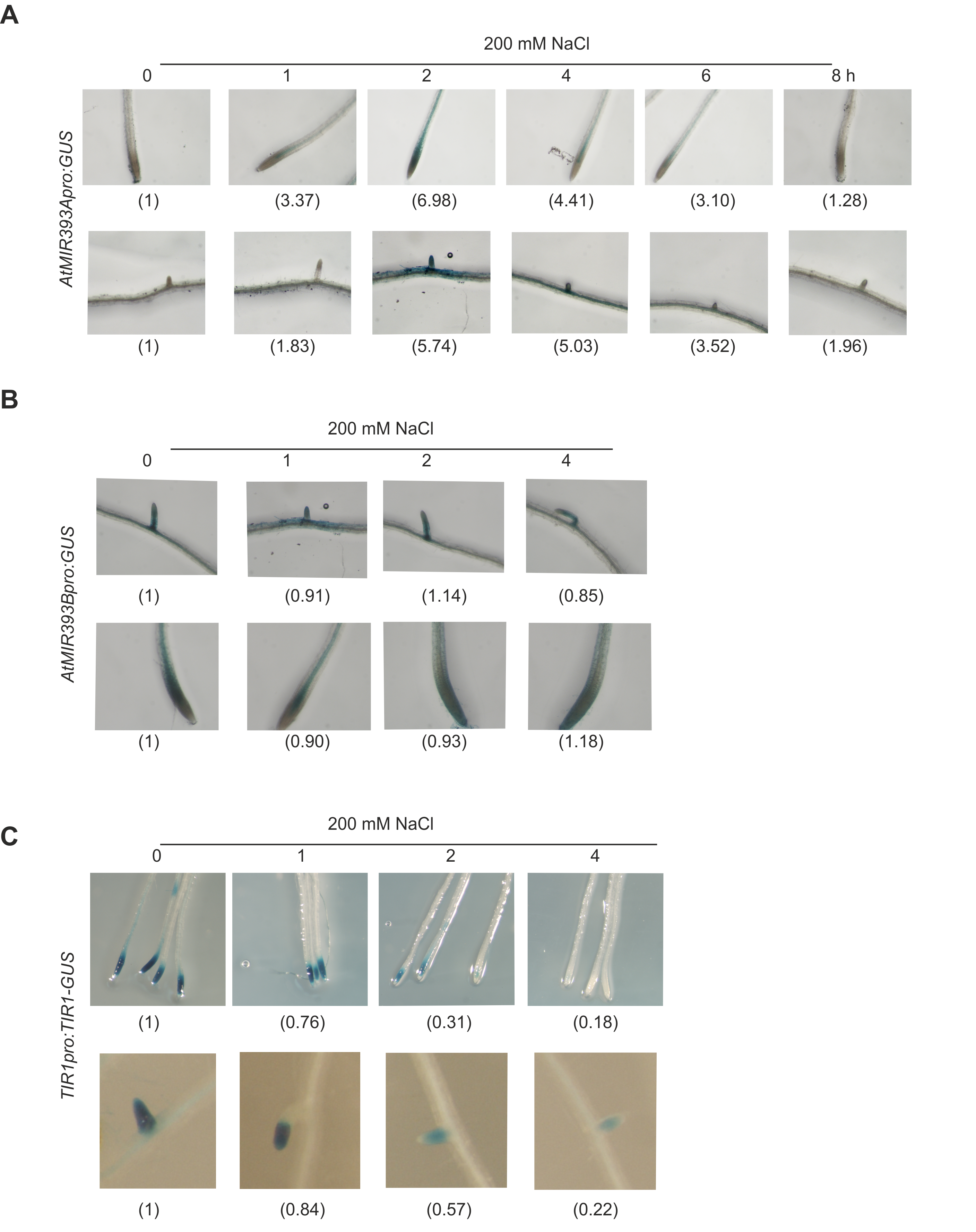

Supplement: Figure S2 — Salinity effect on MIR393Apro:GUS , MIR393Bpro:GUS and TIR1pro:TIR1-GUS seedlings. Seven dpg (A) MIR393Apro:GUS, (B) MIR393Bpro:GUS and (C) TIR1pro:TIR1-GUS seedlings were transferred to liquid ATS medium supplemented with 200 mM NaCl for designated times. Representative photographs of root tips and the GUS signal detected in NaCl treatment relative to control is shown. The control value is arbitrarily set to 1 in each case. (TIF) [file pone.0107678.s002.tif]

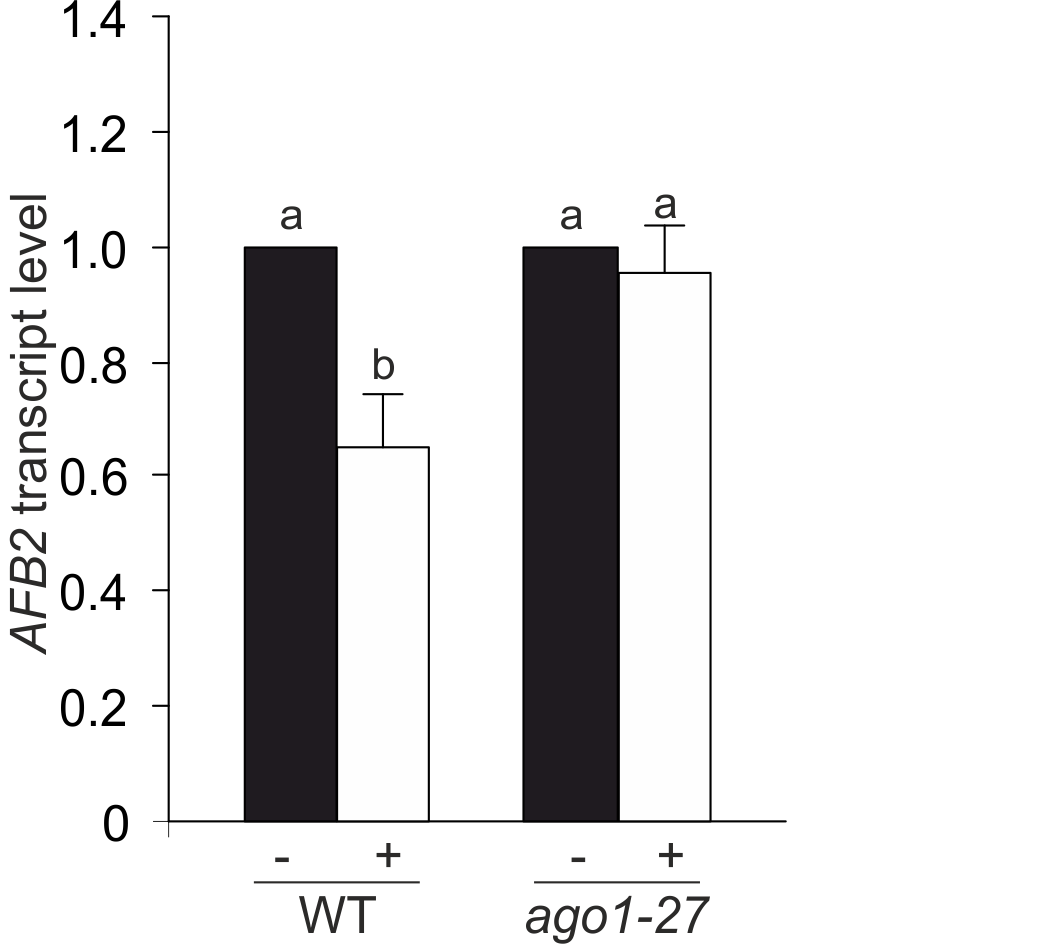

Supplement: Figure S3 — AFB2 relative transcript levels in ago1-27 and WT seedlings upon NaCl treatments. Seven dpg WT and ago1-27 seedlings were subjected to 200 mM NaCl treatment for 4 h. Relative transcript level of AFB2 upon treatment was measured by RT-PCR. The control value is arbitrarily set to 1 in each case. Data are mean values (±SE) of three independent experiments. Different letters indicate a significant difference at P≤0.05 (Tukey test). (TIF) [file pone.0107678.s003.tif]

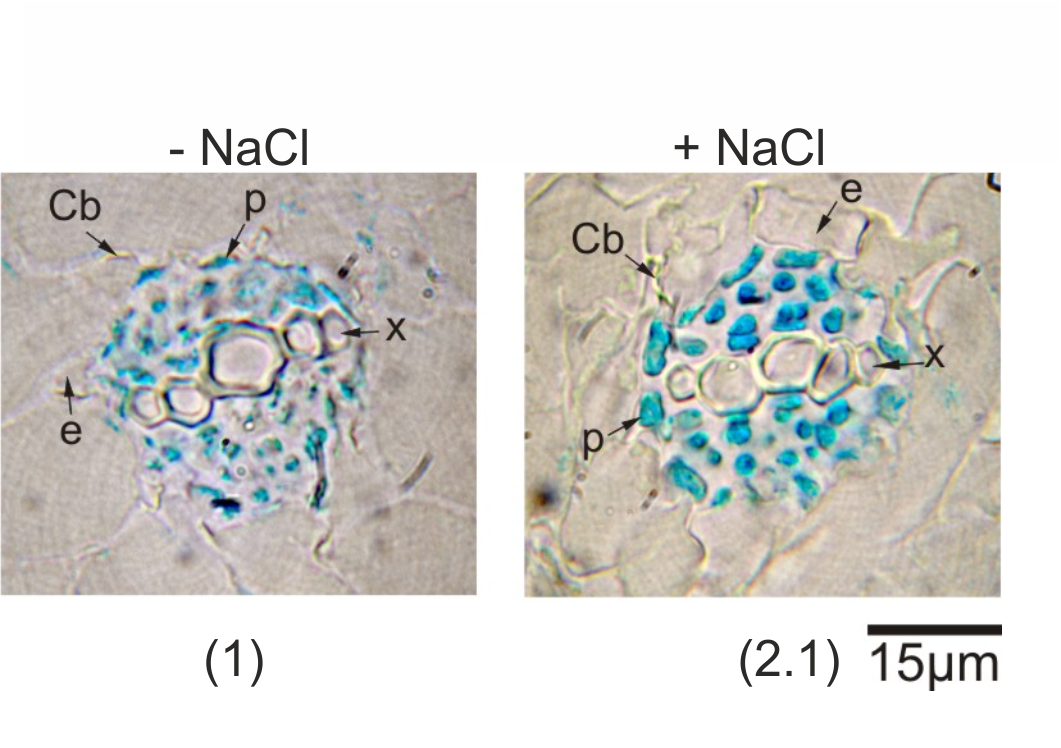

Supplement: Figure S4 — MIR393A promoter activity in cross sections of MIR393A::GUS roots upon NaCl. Seven dpg MIR393Apro:GUS seedlings were transferred to liquid ATS medium supplemented with 200 mM NaCl for 2 h. Seedlings were included in a paraffin matrix (Paraplast) at 60°C and roots were cut into 5 µm sections using a Minot type rotary microtome Zeiss HYRAX M 15. Section were deparaffined with xylene, mounted with Entellan and observed by bright field microscopy in an Olympus CX21 microscope. Images were captured using a digital camera attached to the microscope. e: endodermis; p: pericycle; Cb: Casparian band; x: xylem. The control value of GUS staining is arbitrarily set to 1. Data are mean values of 3 independent experiments (n = 6). (TIF) [file pone.0107678.s004.tif]

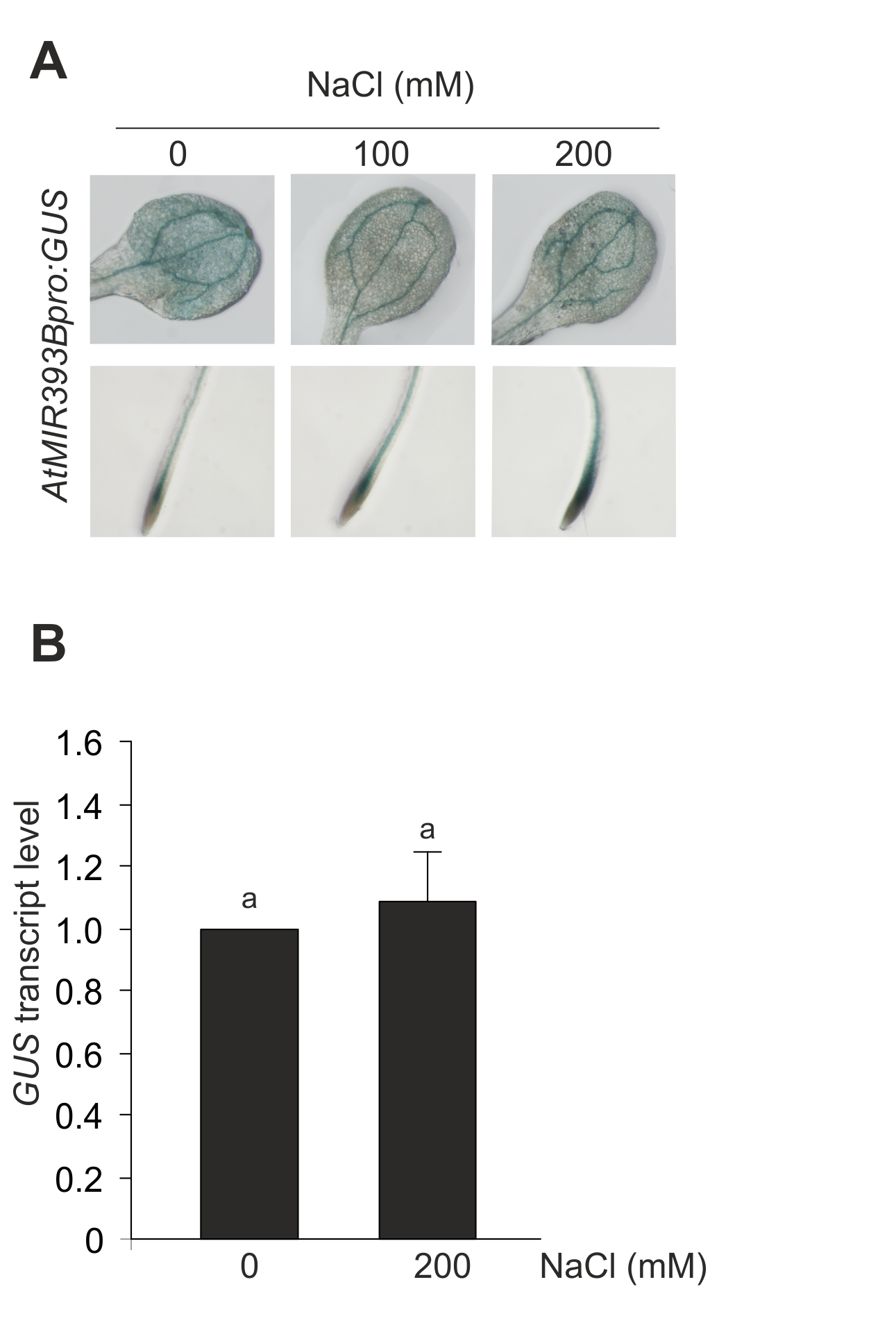

Supplement: Figure S5 — MIR393B promoter activity upon NaCl treatment in AtMIR393Bpro:GUS plants. (A) Seven dpg AtMIR393Bpro:GUS seedlings were transferred to liquid ATS medium supplemented with increasing concentrations of NaCl for 2 h. GUS activity was revealed after incubation with X-Gluc at 37°C. GUS staining in representative leaves and root segments are shown. (B) Relative transcript level of GUS upon 200 mM NaCl treatment as described in (A). The control value is arbitrarily set to 1 in each case. Data are mean values (±SE) of three independent experiments. (TIF) [file pone.0107678.s005.tif]

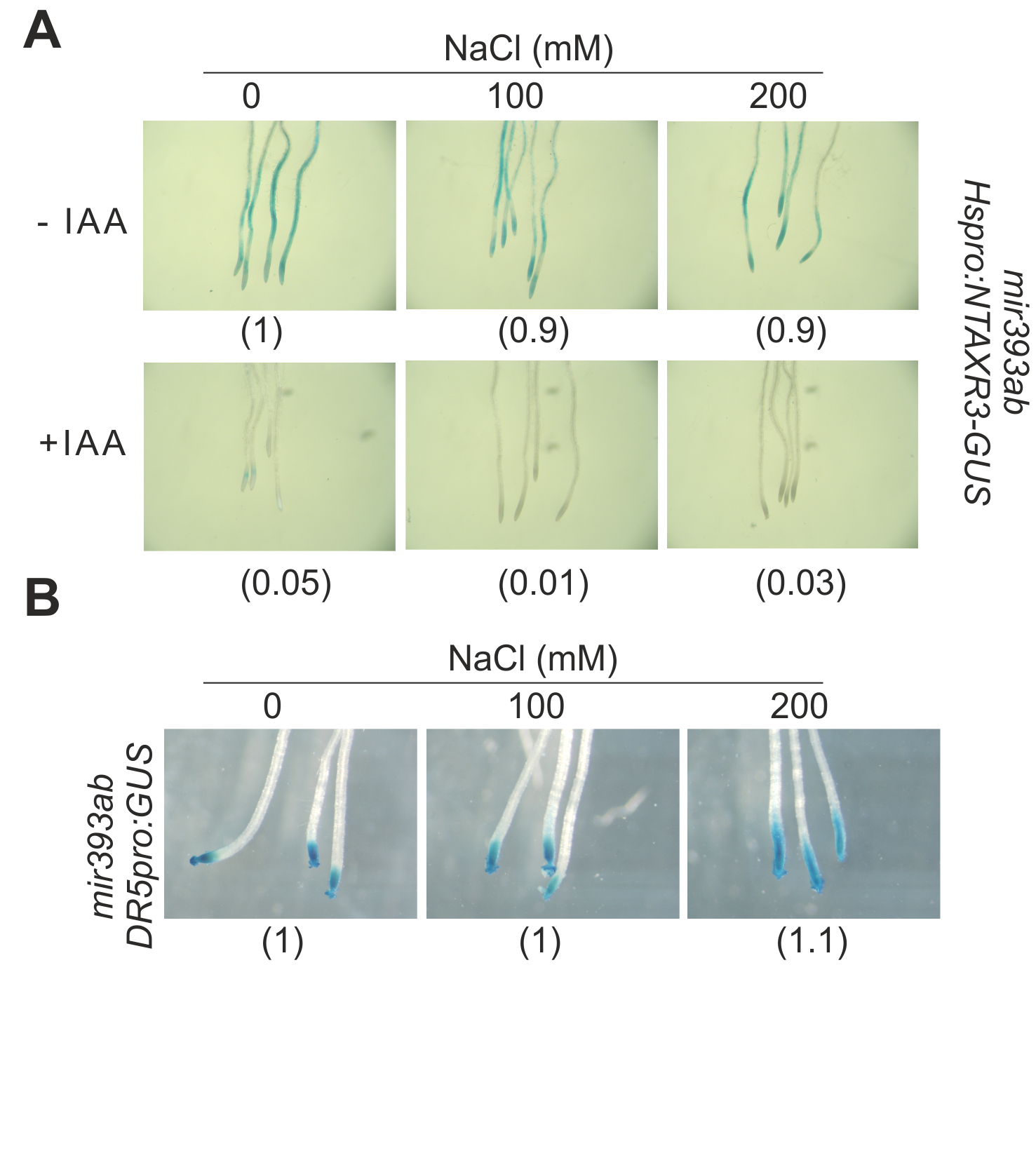

Supplement: Figure S6 — Salinity effect on HSpro:AXR3NT-GUS and DR5pro:GUS in mir393ab background. Seven dpg seedlings were incubated in liquid ATS medium with increasing concentrations of NaCl for 4 h and then subjected to GUS staining. Representative photographs of root tips of mir393ab HSpro:AXR3NT-GUS (A) and mir393ab DR5pro:GUS (B). In (A) seedlings were previously treated for 2 h at 37°C. The control value is arbitrarily set to 1 in each case. Data are mean values of three independent experiments. (TIF) [file pone.0107678.s006.tif]

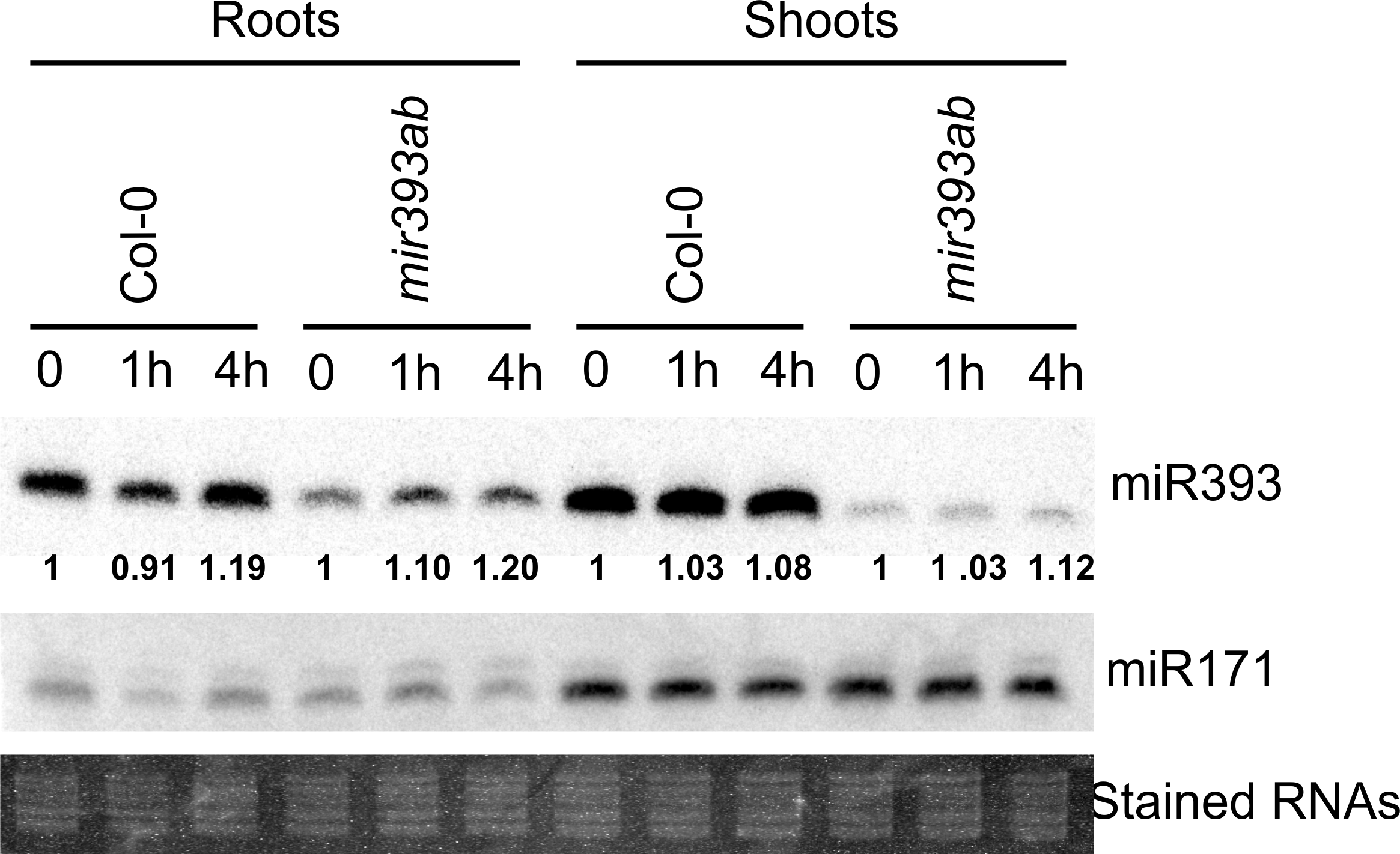

Supplement: Figure S7 — miR393 levels during salinity in roots. Small RNA blot hybridization of RNA (25 µg) from roots and shoots of 7 dpg seedlings treated with 200 mM NaCl for designated times. Probed sRNAs are indicated on the right. The signal detected in mutants relative to control is normalized to signals for the unrelated miR171. The control value is arbitrarily set to 1 in each case. (TIF) [file pone.0107678.s007.tif]

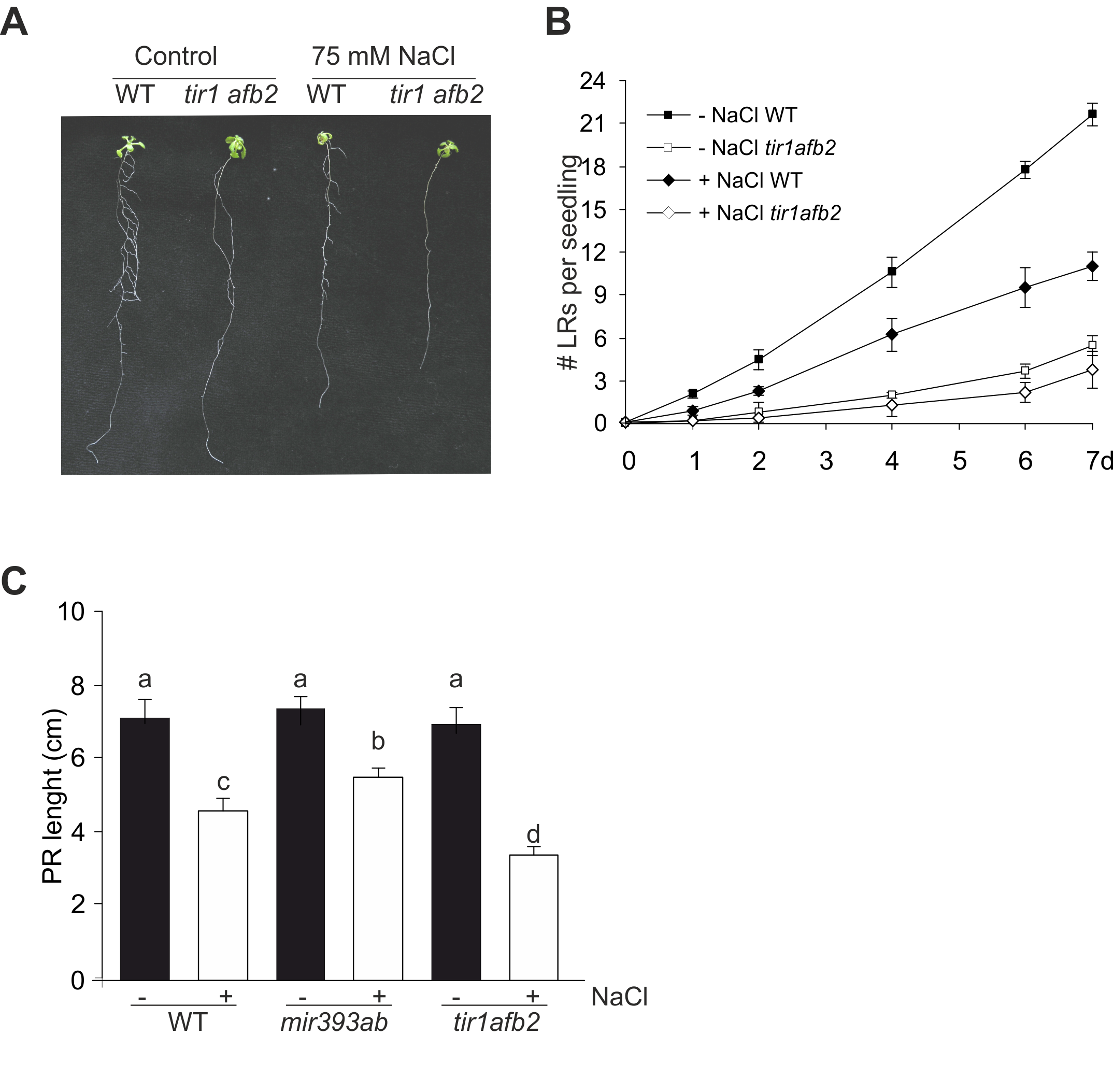

Supplement: Figure S8 — tir1 afb2 and mir393ab root morphological responses. Four dpg WT, mir393ab and tir1 afb2 seedlings were transferred onto ATS medium containing 75 mM NaCl. Representative photographs of tir1afb2 seedlings after 5 d of treatment are shown in (A). LRs were quantified at designed times (B). (C) PR length of WT, mir393ab and tir1afb2 seedlings was measured after 5 d of treatment. Data are mean values (±SE) of three independent experiments. (TIF) [file pone.0107678.s008.tif]

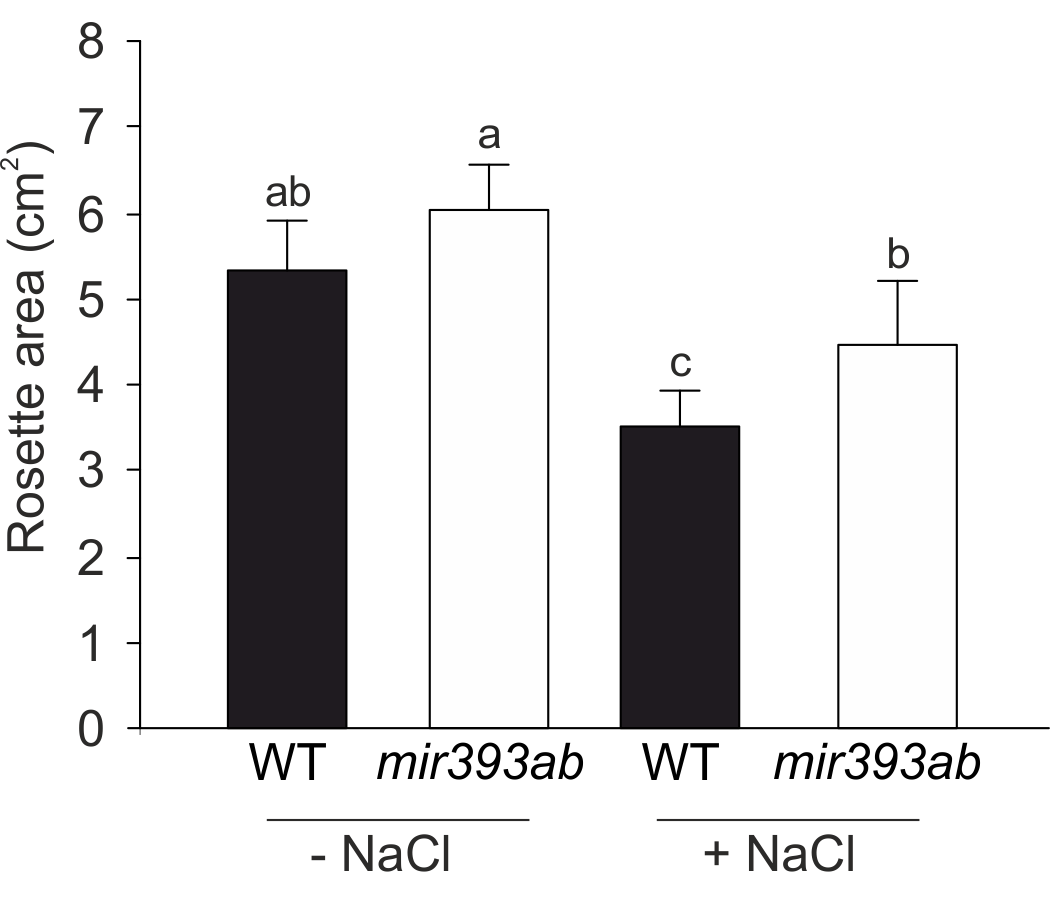

Supplement: Figure S9 — mir393ab morphological response in leaves. WT and mir393ab seedlings were grown in ATS medium supplemented with or without 75 mM NaCl in horizontal position. Rosette area was measured after 12 d of treatment by finding the minimal circle area that contained all leaves. Data are mean values (±SE) of three independent experiments. Different letters indicate a significant difference at P≤0.05 (Tukey test). (TIF) [file pone.0107678.s009.tif]

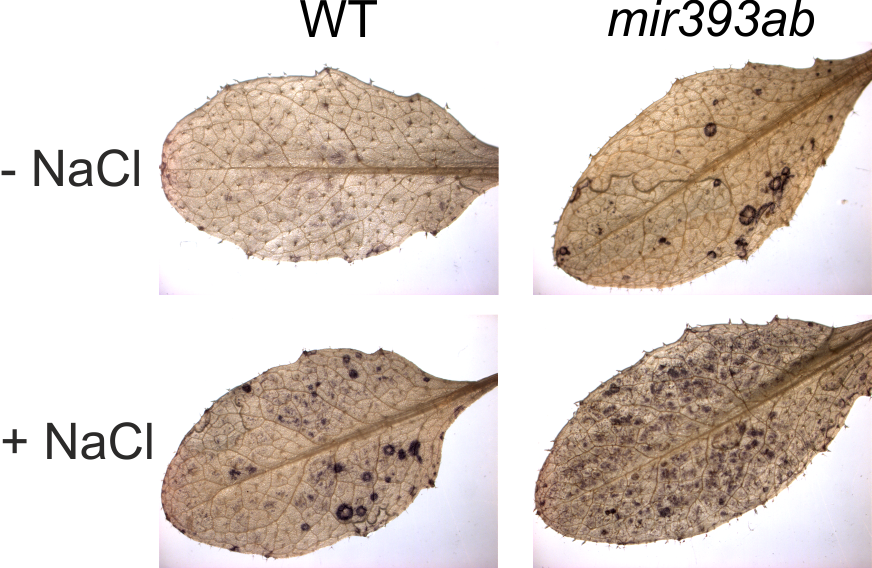

Supplement: Figure S10 — O2 −. level in mir393ab mutant under salinity. Fourteen dpg WT and mir393ab leaves were transferred onto liquid ATS medium supplemented with 100 mM NaCl. After 12 h of initial treatment in situ O2 −. accumulation was detected by NBT staining. Representative photographs are shown. (TIF) [file pone.0107678.s010.tif]

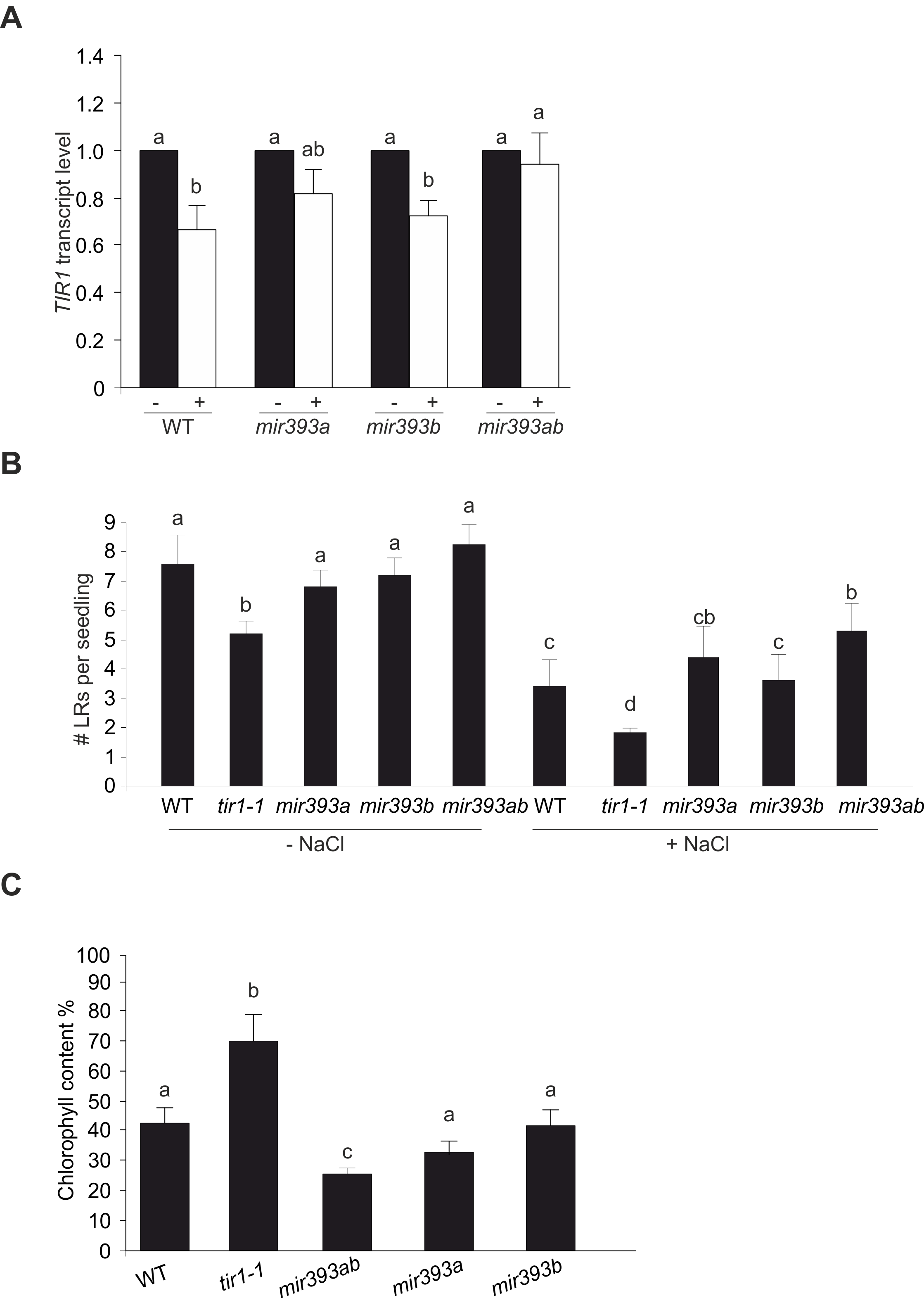

Supplement: Figure S11 — Analysis of single mutants mir393a and mir393b. (A) Seven dpg seedlings were subjected to 200 mM NaCl treatment for 4 h. Relative transcript level of TIR1 upon treatment was measured by RT-PCR. The control value is arbitrarily set to 1 in each case. Data are mean values (±SE) of three independent experiments. (B) Four dpg seedlings were transferred onto ATS medium containing 75 mM NaCl. LR were quantified after 5 d of treatment. Data are mean values (±SE) of three independent experiments. (C) Seven dpg seedlings were treated with 100 mM NaCl for 3 d. Chlorophyll content was measured and expressed as percentage of untreated seedlings. Data are mean values (±SE) of three independent experiments. Different letters indicate a significant difference at P≤0.05 (Tukey test). (TIF) [file pone.0107678.s011.tif]

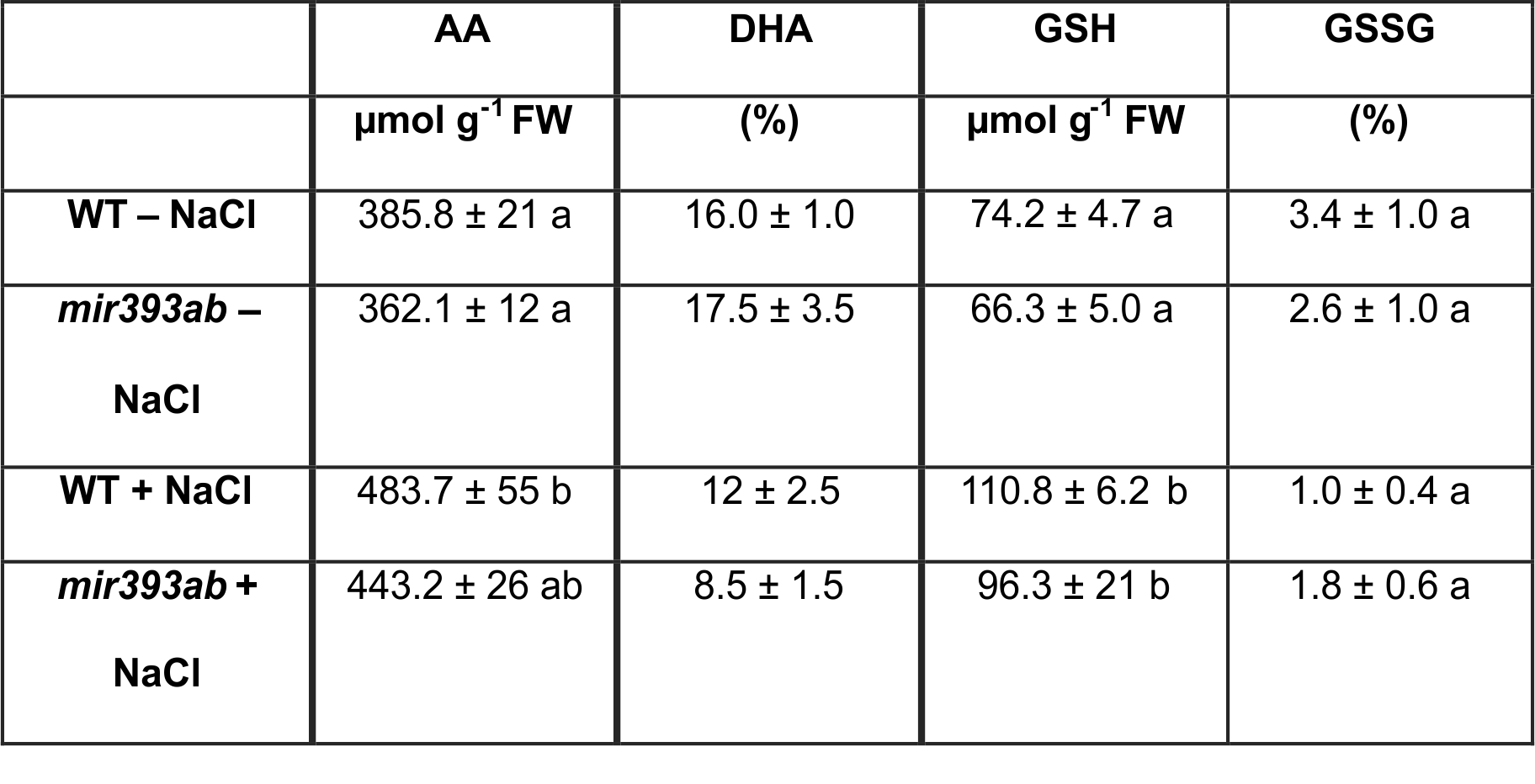

Supplement: Table S1 — Antioxidant levels in WT and mir393ab plants under salinity. Seven dpg WT and mir393ab seedlings were subjected to 100 mM NaCl treatment. After 12 h, AA and GSH levels were quantified. Data are mean values (±SE) of three independent experiments. Different letters indicate significant difference at P≤0.05 (Tukey test). (TIF) [file pone.0107678.s012.tif]
